# The effectiveness of psychosocial interventions on physiological symptoms of menopause: a systematic review and meta-analysis

**DOI:** 10.1186/s12905-026-04563-3

**Published:** 2026-06-08

**Authors:** Kate Robinson, Rebecca Hardy, Melissa Melville, Simone Saidel, Roopal Desai, Aimee Spector

**Affiliations:** 1 Department of Clinical, Educational and Health Psychology, University College London, London, UK; 2 School of Sport, Exercise and Health Science, Loughborough University, Loughborough, UK; 3 Research and Development Department, North East London NHS Foundation Trust Goodmayes Hospital, Ilford, UK

**Keywords:** Psychosocial interventions, Menopausal symptoms, Physiological symptoms, Randomised controlled trials, Meta-analysis, Menopause

## Abstract

**Background:**

Menopause is a transitional life stage marked by the end of menstruation, during which approximately 80–90% of women experience persistent symptoms such as vasomotor symptoms, musculoskeletal pain, fatigue, and sleep disturbance. While menopausal hormone therapy is effective for some symptoms, non-pharmacological psychosocial interventions are increasingly recommended as alternatives or adjuncts. This review evaluates the efficacy of psychosocial interventions for managing physiological menopausal symptoms.

**Methods:**

Six databases were systematically searched from inception to October 2024 for randomised controlled trials evaluating psychosocial interventions for menopausal women with physiological symptoms. Outcomes were grouped into nine categories, including sleep quality, insomnia, pain, fatigue, urogenital symptoms, sexual functioning, and vasomotor symptoms (classified as frequency, bothersomeness, and severity). Primary analyses used post-intervention data, with sensitivity analyses based on change scores. Effect sizes were expressed as Hedges’ g. The protocol was preregistered on PROSPERO, CRD42024572869.

**Results:**

28 randomised controlled trials involving 2,887 women were included, of which 24 were included in the meta-analysis and four synthesised narratively. Psychosocial interventions produced medium-to-large reductions in the bothersomeness of hot flushes and night sweats at short-term follow-up (Hedges’ g = − 0.60 to − 0.87) and medium-term follow-up (g = − 0.50 to − 0.77), while effects on symptom frequency and severity were smaller. Significant improvements were observed in sleep quality (short-term: g = − 0.77 to − 1.04; medium-term: g = − 0.46) and insomnia (short-term: g = − 1.77 to − 2.48; medium-term: g = − 1.56 to − 1.79). Psychosocial interventions did not demonstrate improvements on sexual functioning or urogenital symptoms. Intervention dose varied across studies, and retention rates were high (mean = 86.7%), indicating good feasibility.

**Conclusion:**

Psychosocial interventions, particularly cognitive behavioural therapy, were associated with improvements in menopausal symptoms, with the strongest and most consistent effects observed for vasomotor symptom bothersomeness and sleep outcomes. These findings support psychosocial approaches as valuable non-pharmacological options, either alone or alongside pharmacological treatments. Future research should focus on tailoring interventions to individual needs, assessing whether benefits are maintained long-term, and examining whether effectiveness varies across different stages of menopause.

**Supplementary Information:**

The online version contains supplementary material available at 10.1186/s12905-026-04563-3.

## Introduction

Menopause marks the end of menstruation and may occur naturally or as a result of medical treatment [50]. The menopause transition includes the peri-menopausal, menopausal, and post-menopausal stages, with symptoms often persisting for several years (Gatenby et al., [21]; [27]. Symptoms result from hormonal shifts and affect 80–90% of women [5], with variation across individuals, cultures, and ethnicities [29]. Vasomotor symptoms (VMS) such as hot flushes and night sweats are commonly reported [56], alongside urogenital changes, sleep difficulties, sexual dysfunction, fatigue, and musculoskeletal issues [48]. Such symptoms can impair quality of life and contribute to health risks [25].

Menopausal hormone therapy (MHT) is effective for managing vasomotor and urogenital symptoms [7], but it is not suitable for all women (Poggio et al. 54, and some women prefer psychological rather than pharmacological approaches [30]. Clinical guidelines recommend psychosocial interventions, particularly Cognitive Behavioural Therapy (CBT) which helps women reframe thoughts and behaviours during menopause (NICE [51]). Other approaches include mindfulness [8], relaxation [18], and Acceptance Commitment Therapy [47].

Existing reviews have tended to focus narrowly on the treatment of hot flushes [55]. Others have been limited to face-to-face interventions or have combined physical and psychological outcomes, making it difficult to determine the effects of psychosocial interventions across a broader range of physiological symptoms [65]. In the present review, physiological symptom domains included sleep quality, insomnia, fatigue, pain, urogenital symptoms, sexual functioning, and VMS. Cognitive symptoms such as ‘brain fog’ were not included within the scope of this review.

Digital, self-help, and third-wave psychosocial approaches, such as Acceptance Commitment Therapy, remain underrepresented in the literature [28]. This review addresses these gaps by evaluating various psychosocial interventions across multiple physiological symptom domains and delivery formats. The primary aim was to assess effects on physiological symptoms, with a secondary aim of examining retention rates as an indicator of feasibility.

## Methods

The review was registered on PROSPERO in July 2024, CRD42024572869 and conducted in accordance with PRISMA 2020 guidelines (Page et al. [53]).

### Eligibility

Inclusion and exclusion criteria were developed with reference to existing systematic reviews in this area [57, 61]. For the purpose of this review, structured psychosocial interventions were defined as non-pharmacological interventions with a clearly specified mechanism of action (e.g. cognitive, emotional, or behavioural change processes). Eligible interventions included CBT, mindfulness-based interventions, acceptance and commitment therapy (ACT), relaxation-based interventions, and guided self-help.

#### Inclusion criteria

1. Randomised Controlled Trials (RCTs), including feasibility and pilot studies.
2. Peer-reviewed publications written in English.
3. Enrolled female participants experiencing menopausal transition (either natural or treatment-induced), with menopausal status determined by clinical diagnosis, reproductive aging stage (e.g. peri- or post-menopause), or participant self-report of symptoms consistent with the menopausal transition.
4. Evaluated a structured psychosocial intervention, as defined above, delivered in any format (group or individual; online or face-to-face; facilitated or self-help; tailored to the menopause or not).
5. Reported at least one validated outcome measure assessing physiological menopausal symptoms.

#### Exclusion criteria

- Evaluated primarily educational, exercise-based, or alternative therapy interventions (e.g. acupuncture).
- Targeted chronic physical conditions not related to menopause, even when conducted in women of menopausal age.
- Reported only multidimensional outcome measures combining physical and psychological symptoms, where physical symptom data could not be extracted or analysed separately.

#### Control group

Control conditions were not restricted and included active controls, waitlist controls, and treatment-as-usual. This approach was adopted to reflect the heterogeneity of comparators used in psychosocial intervention trials for menopausal symptoms and to maximise inclusivity, given the limited number of RCTs in this area.

### Information sources and search strategy

A simultaneous search was executed across PsycINFO, MEDLINE, Embase, CINAHL, Web of Science and Cochrane Review, from inception to October 2024, including OVID, EMSCOhost, Web of Science, and COCHRANE interfaces. Grey literature was retrieved through Google Scholar. Full search syntax is provided in Supplementary Materials.

### Study selection

**Fig. 1:**
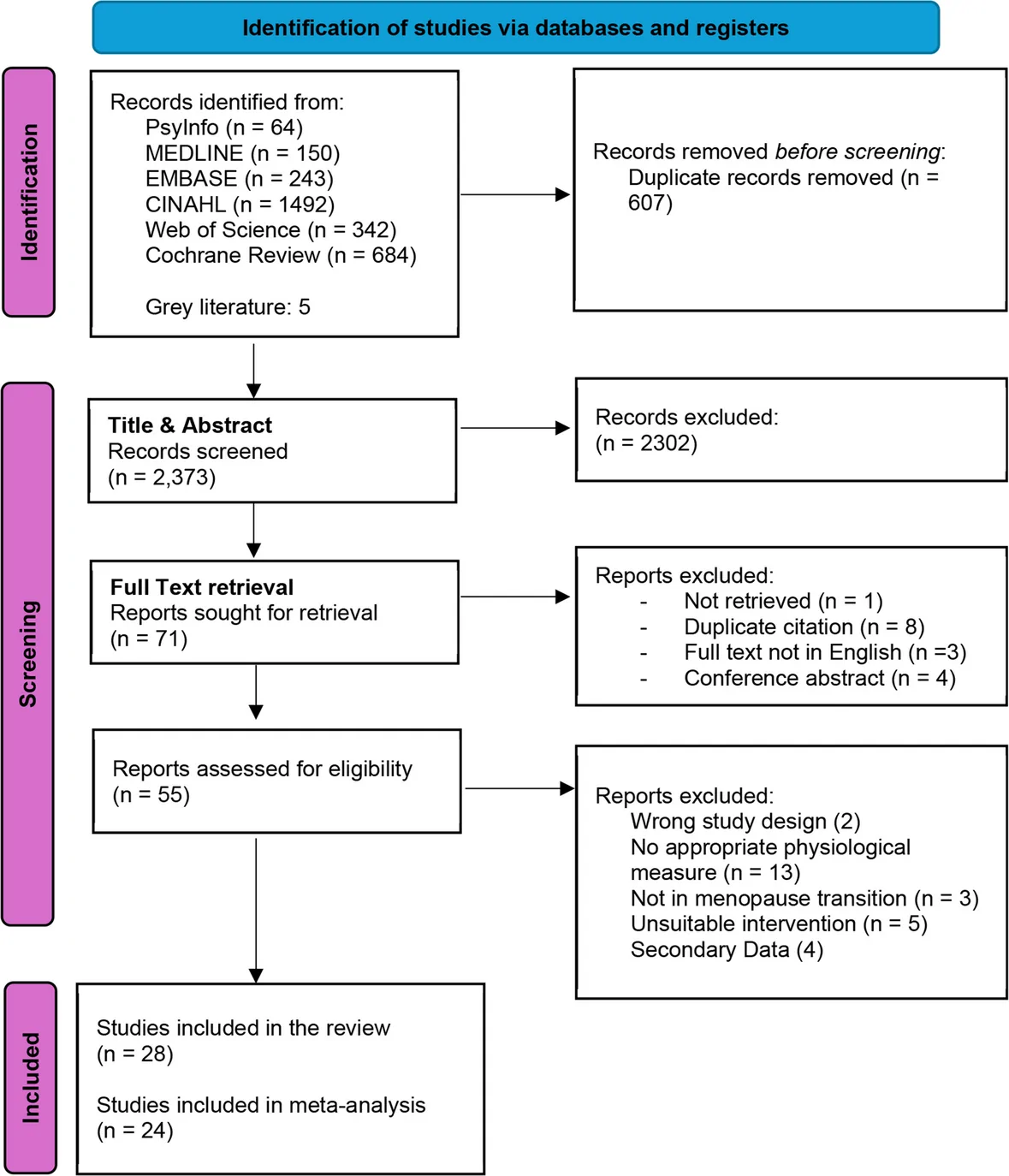
*Prisma diagram depicting the process of study selection. *(Page et al. [53])

One reviewer (KR) screened all titles and abstracts and conducted full-text screening with a second reviewer (SS) independently checking 10% at both stages. Disagreements were resolved through consultation with another member of the team. Studies were screened according to the above criteria. The PRISMA flow chart (Fig. 1) summarises the screening and selection process.

### Data extraction

A summary table was used to extract study and intervention characteristics (Tables 1 and 2), including intervention type, session duration, outcome measures, and mean scores with standard deviations for intervention and control groups at pre- and post-test. One reviewer completed the extraction also recording retention rates to assess feasibility.

**Table 1:** Characteristics of Included Studies

**Author, date**	**Country**	**Description of participants: Total N included, Mean Age in years (SD/Range), Demographics (e.g., Ethnicity, Education, Employment/Household Income)**	**Menopause Type**	**Control group**	**Retention Rate**	**Follow-up periods**	**Domain Measures and Outcome measures**
Abdelaziz et al 2022 [1]	Saudi Arabia	*n*= 80 Int: *Individual CBT:* 53.90 (4.14); education (17.5% secondary and above); 27.5% employed Con: 52.23 (4.31); education (17.5% secondary and above); 17.5% employed.	Natural	Non-active	81.63%	Week 6*	Sleep quality: PSQIInsomnia: ISI
Anderson et al 2015 [2]	Australia	*n* = 51 Int: *Wellness Program*: 48.5 (5.9); education (University 46.3%); Employment (full time, 66.7%); Income ('High' (>80k AUD 63%) Con: 49.8 (6.5); education (university 15.4%); employed (full time 65.4%), income (High ' (>80k AUD) 60%)	Treatment induced	Active Control:Booklet	92.70%	Week 6*, 12	Vasomotor severity: GCS Vasomotor subscaleSleep Quality: PSQISexual functioning: GCS Sexual subscale
Atema et al 2019 [3]	Netherlands	*n* = 254 Int: *Self-guided CBT:* 47.5 (5.14), education (high school 100%); employed (78.8%)*Self-help CBT:* 47.7 (5.73), education (high school 97.6%) ; employed (72.9%) Con:47.0 (5.50), education (high school 100%); employed (82.1%)	Treatment-induced	Non active (waitlist)	92.91%	Week 10*, week 24	Hot Flush bothersomeness: HFRS problem rating subscale (distress, interference, overall problem). Sleep Quality: GSQSHFRS frequency subscale: Hot flush frequencySexual functioning: SAQ discomfortVitality: SF-36 subscalePain: SF-36 subscale
Augoulea et al 2021 [4]	Greece	*n* = 63 Int: *Group Stress Management Program*: 57.70 (6.89) Con: 56.52 (4.73)	Natural	Active control: Verbal advice and weekly follow up call	96.80%	Week 8*	Vasomotor severity: GCS Vasomotor subscaleSexual functioning: GCS Sexual subscaleVitality: SF-36 subscalePain: SF-36 subscale
Ayers et al 2012 [6]	UK	*n* = 140 Int: *Group CBT:* 53.73 (5.9), ethnicity (White 82 %), education (degree 53 %), employed (76 %)*Self-help CBT:* 51.70 (4.4), ethnicity (White 87 %), education (degree 55 %), employed (81 %). Con: 53.87 (5.7), ethnicity (White 78 %), education (degree 62 %), employed (65 %)	Natural	Non active (no intervention)	92.14	Week 6*, 26	Hot Flush bothersomeness: HFRS problem rating subscale (distress, interference, overall problem) Sleep quality: WHQ subscaleVitality: SF-36 subscalePain: SF-36 subscale
Carmody et al 2011 [8]	USA	*n* = 110 Int: *Group Mindfulness Based Intervention:* 52.93 (3.29); ethnicity (White 98.2%)education (college and above 91.1%), employment(full time 63 %) Con: 53.8 (4.4); ethnicity (White 100%), education (college and above 92.2%), employment (full time 67.9 %)	Not stated	Non active(waitlist)	90.90%	Week 8*, 3 months	Insomnia: WHIIRSHF Bothersomeness: HFRS problem rating subscale
Drake et al 2019 [13]	*USA*	*n* = 150 Int: *Individual CBT:* 55.32 (5.90), ethnicity (White 48%)*Individual Sleep Restriction Therapy: *56.76 (5.39), ethnicity (White 56%) Con: 57.24 (5.55), ethnicity (White 52%)	Both	Active Control (SleepHygiene Education)	100.00%	Week 6*, 6 months	Insomnia: ISI
Duijts et al 2012 [14]	Netherlands	n = 212 Int: *Group CBT:* 48.2 (5.7); education (college 33%); Employed (full time 21.1%) Con.: 47.8 (6.0); education (college 36.9%); Employed (full time 18.4%)	Treatment induced	Non active(waitlist)	80.57%	Week 10*, 24	Hot Flush bothersomeness: HFRS problem rating subscale (distress, interference, overall problem)HF/NS Frequency: HFRS frequency rating subscaleUrogenital: BFLUTSVitality: SF-36 subscalePain: SF-36 subscale
El‐Monshed et al 2024 [15]	Egypt	*n* = 98 Int: *Group CBT:* age (40-<50, 55.8%); education (university 30.2%); Employed (48.8%) Con.: age (40-<50, 57.8%); education (university 26.7%), Employed (42.2%)	Natural	Active control: TAU, General health advice	89.70%	Week 7*	Sleep quality: PSQIInsomnia: ISI
Farsani et al 2021 [16]	Iran	*n*= 46 Int: *Group CBT:* 51.41 (3.00); Education (High school, 40.91%); Employment (Homemaker 81.82%) Con.: 52.35 ± 3.48; Education (high school, 39.13%); Employment (Homemaker 91.30%)	Not stated	Active control: TAU	97.80%	Week 3, 6, 10*	Sleep quality: PSQIInsomnia: ISI
Fenlon (1999) [17]	UK	*n* = 24 Int: *Individual Relaxation Therapy:* 49 (median). Con.: 48 (median). All European and 'Caucasian'. Most from a middle-class area.	Treatment induced	Non active(waitlist)	66.70%	Week 4*	Hot Flush bothersomeness: HFRS problem rating subscale (distress, interference, overall problem)
Fenlon et al 2008 [18]	UK	*n* = 150 Int: *Individual Relaxation Training:* 54.9 (Median), Ethnicity (Caucasian 93%) Con: 55.4 (Median), Ethnicity (Caucasian 97%)	Treatment induced	Active control: Discussion with Nurse	64.67%	Week 4*, 3 months	Hot Flush bothersomeness: HFRS problem rating subscale (distress, interference, overall problem)HF frequency: Hot flush frequency
Fenlon et al 2020 [19]	UK	*n* = 127 Int: *Group CBT:*53.5 (9.78); ethnicity (white, 96.7%); Education (+16 years of age 64.4%; Employment (56.7%) Con: 55.20 (10.19); ethnicity (white, 95.4); Education (+16 years of age 46.2%; Employment (60.6%)	Treatment induced	Non active(waitlist)	97.69%	Week 9*, 26	Hot Flush bothersomeness: HFRS problem rating subscale (distress, interference, overall problem). HF/NS Frequency: HFRS frequency rating subscaleSleep Quality: PSQI
Garcia et al 2018 [20]	Brazil	*n* = 30 Int: *Individual Mindfulness Based Intervention*: 55.16 (6.05) Con: 56.68 (4.01)	Natural	Active control: Talking	86%	Week 8	Sleep quality: PSQIInsomnia: ISI
Gordon et al 2021 [23]	Canada	*n*=104 Int: *Group Mindfulness Based Intervention*: 48.7 (3.0); Ethnicity (Caucasian 90%) Con.: 48.7 (3.7); Ethnicity (Caucasian 89%)All participants had either some university education or a bachelor’s degree and had an approximate income of $90,000 – 113,000+"	Not specified	Non active(waitlist)	N/A	Week 8*, 4 and 6 months	Sleep quality: PSQI
Green et al 2019 [24]	*Canada*	*n* = 71 Int: *Group CBT:* 53.27 (3.69); Ethnicity (White 91.9%); Employed (full time 64.9%) Con.: 52.88 (4.39); Ethnicity (White 85.3%); Employed (full time 38.2%)	Both	Non active(waitlist)	61.11%	Week12*, 3months	Vasomotor severity: GCS Vasomotor subscaleSleep quality: PSQISexual functioning: FSFI
Hardy et al 2018 [26]	UK	*n* = 124 Int: *Individual CBT: *54.04 (3.17); ethnicity (WhiteBritish 70.0 %); education (degree 61.6 %), employment (full time, 76.7%) Con.: 54.10 (3.53); ethnicity (White British 71.4 %); education (degree 65 %); employment (full time, 89.1%)	Both	Non active(waitlist)	85.48%	Week 6*, 20	Hot Flush bothersomeness: HFRS problem rating subscale (distress, interference, overall problem). HF Frequency: HF frequency rating subscaleSleep quality: WHQ subscale
Hsiu-Chin et al 2015 [32]	China	*n* = 59 Int: *Group Sleep-based Intervention:* 49.52 (4.03), Employed (79.3%), education (college, 20.7%) Con: 49.57 (3.41), Employed (93.3%), education (college, 13.3%)	Natural	Active: TAU and greeting calls	93.60%	Week 5, 8*	Sleep quality: PSQI
Hummel et al 2017 [33]	Amsterdam	*n* = 169 Int: *Individual CBT: *51.6 (7.7); Education (higher ed, 77.1%) Employed (77.4%) Con.: 50.5 (6.8); Education (higher ed, 86.9%) Employed (81.2%)	Treatment induced	Active Control:Booklet	89.30%	Week 10*, 20-24	Sexual functioning: FSFIVitality: SF-36 subscalePain: SF-36 subscale
Jokar et al 2016 [37]	Iran	*n* = 129 Int: Group Relaxation Training: 55.23 (4.88); Education (diploma 39.5%); Employment (housewife 86%) Con: 54.78 (3.55); Education (diploma 14.28%), Employment (housewife 88.1%)	Natural	Active Control (Placebo)	100%	Week 4*	Sleep quality: PSQI
Keefer & Blanchard (2005) [38]	USA	*n* = 19 Int: *Group-CBT* Ethnicity (white 86%) for total sample	Not stated	Non active(waitlist)	N/A	Week 8*	Hot Flush bothersomeness: HFRS problem rating subscale (distress, interference, overall problem).HF/NS Frequency: Frequency subscale
Lindh-Åstrand & Nedstrand (2013) [39]	Sweden	*n* = 60 Int: *Group Applied Relaxation*:54.0 (5.7) Con.: 56.0 (5.1). Five women were retired, and the remainingwomen were part or full daytime workers.	Both	Non active(waitlist)	95%	Week 12*, 3 months	HF/NS bothersomeness: WHQ subscaleSleep quality: WHQ subscale
Mann et al 2012 [42]	UK	*n* = 96 Int: *Group-CBT:* 53.16 (8.10); ethnicity (White 89%), education (Beyond 16 years of age 64 %), employed (64 %). Con.: 54.05 (7.76); ethnicity (White 82%), education (beyond 16 years of age 67 %), employed (65 %)	Treatment induced	Active Control:Nursepsycho-ed &breathing	91.67%	Week 9*, 26	Hot Flush bothersomeness: HFRS problem rating subscale (distress, interference, overall problem). HF/NS frequency: HF/NS frequency subscaleSleep Quality: WHQ subscale Vitality: SF-36 subscalePain: SF-36 subscale
McCurry et al 2016 [45]	USA	*n* = 106 Int: *Individual CBT:*55.0 (3.5), ethnicity (white 92.5%), education (college grad 83%) Con:54.7 (4.7), ethnicity (white 90.6%), education (college grad 73.6%)	Natural	Active Control:Medical education	76.40%	Week 8*, 24	Sleep quality: PSQIInsomnia: ISI
Mojtehedi et al 2022 [46]	Iran	*n* = 66 Int: *Group Mindfulness Based Intervention:* 56.00 (3.21); Education (High school diploma 78.78%); Employment (Housewife 84.85%) Con: 56.59 (4.25); Education (High school diploma 75.76%); Employment (Housewife 81.83%)	Natural	Active control: TAU and Placebo	86.40%	Week 8*, 16	Sexual functioning: FSFI
Monfaredi et al 2022 [47]	Iran	*n* = 86 Int: *Group Mindfulness Based Intervention:*: 54.87 (4.49); Education (university 4.7 %); Employment (housewife 88.4 %) Con.: 54.75 (4.56); Education (university 14 %); Employment (housewife 83.7 %)	Natural	Active control: TAU	100%	Week 8*	Sleep quality: PSQI
van Driel et al 2019a, b [61, 62]	Netherlands	*n* = 66 Int: *Group Mindfulness Based Intervention:* 47.0 (5.0); education (Higher education 32.4%), Employment (part-time 55.9 %). Con.: 48.5 (5.4); education (higher education 54.8 %), Employment (part-time 64.5 %)	Treatment-induced	Active Control:TAU with specialistnurse	72.73%	3 months*, 6 and 12 months	Sexual functioning: FSFI
Wong et al 2018 [64]	China	*n* = 197 Int: *Group Mindfulness Based Intervention:* 51.9 (3.0); education (secondary school 70.4 %), employment (housewife 46.9 %) Con.: 52.1 (3.2); education (secondaryschool 67.7 %), employment (housewife 45.5 %)	Natural	Active control: Menopause education	N/A	Week 8*, 3 and 6 months	Vasomotor Severity: GCS subscaleSexual functioning: GCS subscale Urogenital: GCS subscale

*BFLUTS* Bristol Female Lower Urinary Tract Symptoms, *GCS* Green climacteric scale, *FSFI* Female sexual function index, *GSQS* Groningen Sleep Quality Scale, *HF/NS* Hot flush/Night Sweat. *HFRS* Hot Flush Rating Scale, *ISI* Insomnia severity scale, *SAQ* Sexual Activity Questionnaire, *TAU* Treatment as usual, *PSQI* Pittsburgh Sleep Quality Index, *WHQ* Women’s Health Questionnaire, *WHIIRS* Women's Health Initiative Insomnia Rating Scale

*used for post-intervention analysis

**Table 2:** Summary of Intervention Characteristics

**Reference**	**Content of Intervention**	**Individual or Group**	**Facilitated or Self-directed**	**Face-to-face/Telephone/Digital/Self-help**	**Intervention tailoring**	**Individual Tailoring**	**Number, Length and Frequency of Intervention**	**Approx. Total Contact Hours**	**Intervention Facilitator**
CBT interventions (*n* = 14)									
Abdelaziz et al 2022 [1]	Modules focused on psychoeducation, cognitive intervention, behavioural intervention including exercises for sleep	Individual	Facilitated	Online/self-help + brief weekly phone calls	CBT for sleeping difficulties in menopausal women	No	6 sessions, weekly. Length not specified	6 h + 3 h homework	Study researcher
Atema et al 2019 [3]	Psycho-ed. on HFNS & CBT for menopause. Included relaxation, activity scheduling, problem solving, stress management	Individual	Self- directed	Online/self help	CBT for menopausal symptoms in breast cancer survivors	No	6 × 1 h, weekly + homework	6 h + 3 h homework	Trained medical social workers and psychologists
Ayers et al 2012 [6]	Psycho-ed, stress management, paced breathing, and CBT, including individual goal setting and homework	Group and individual	Both	F-2-F & Self- help	CBT for reducing problematic menopausal hot flushes and night sweats	No	*Group:* 4 × 2 h, weekly + homework. *Self-help:* an introductory session, one guiding telephone call + homework	Group: 8 h + homework	Clinical psychologist
Drake et al 2019 [13]	Behavioural strategies (sleep restriction and stimulus control), Cognitive restructuring for sleep-related thoughts, Relaxation techniques	Individual	Facilitated	F-2-F	CBT for chronic insomnia in postmenopausal women	No	6 × 1 h, weekly	6 h	Registered nurse trained in behavioural sleep medicine
Duijts et al 2012 [14]	Psycho-ed on menopause health changes, relaxation techniques, identify hot flush triggers, problem solving and stress management	Group	Facilitated	F-2-F	CBT and physical exercise for menopausal symptoms in patients with breast cancer	No	6 × 1.5 h, weekly + 6-month booster of 1.5 h	9 h in 6 weeks + 6-month booster	Clinical psychologist and clinical social workers
El‐Monshed et al 2024 [15]	Sleep hygiene, relaxation techniques, psychoeducation and cognitive restructuring	Group	Facilitated	F-2-F	Not Reported	Not reported	7 × 1 h, weekly sessions + homework	7 h + homework	Principal investigator and last author trained in CBT
Farsani et al 2021 [16]	Sleep education, stimulus control, sleep restriction, relaxation training and changing maladaptive sleep beliefs	Group	Facilitated	F-2-F	Not Reported	Not reported	6 × 1 h, weekly	6 h + home practice	Researcher trained in CBT-i
Fenlon et al 2020 [19]	Psycho-ed on CBT for menopause, stress & anxiety, CR, exposure, relaxation and sleep hygiene	Group	Facilitated	F-2-F	CBT for hot flushes and night sweats in women with breast cancer	No	6 × 1.5 h, weekly + homework	9 h in 6 weeks	Breast cancer nurse
Green et al 2019 [24]	Psycho-ed on menopause health changes, CR, activity scheduling, exposure, relaxation, Sleep hygiene and CBTi techniques	Group	Facilitated	F-2-F	CBT for menopausal symptoms	No	12 × 2 h, weekly + homework	24 h + homework	PhD-level licensed clinical psychologist and graduate-level psychology trainee
Hardy et al 2018 [26]	Psycho-ed on menopause, stress management, relaxation, CR & activity scheduling, sleep hygiene and goal setting	individual	Self-directed	Self-help	CBT for working women with problematic hot flushes and night sweats	No	Self-help—Intervention frequency not specified	Hours not specified in 4 weeks	n/a
Hummel et al 2017 [33]	Psychoeducation, cognitive restructuring, sensate focus exercises, communication training, mindfulness, and body image work	Individual	Both	Online/self help	CBT for improving sexual functioning of breast cancer survivors	Yes	20 weekly sessions	Hours not specified	Four female psychologists or sexologists
Keefer & Blanchard (2005) [38]	Psycho-ed on menopause & applied relaxation for mgmt. of HFNS. Relaxation techniques	Group	Facilitated	F-2-F	CBT for menopausal hot flushes	No	8 × 1.5 h, weekly	12 h	Therapist/researcher
Mann et al 2012 [42]	Psycho-ed on CBT for menopause, stress & anxiety, CR, exposure, relaxation and sleep hygiene	Group	Facilitated	F-2-F	CBT for women who have menopausal symptoms after breast cancer treatment	No	6 × 1.5 h weekly + homework	9 h + homework	Clinical psychologist and assistant psychologist
McCurry et al 2016 [45]	Sleep restriction, stimulus control, sleep hygiene education, cognitive restructuring, and behavioural homework	Individual	Facilitated	Telephone	CBT for insomnia in perimenopausal and postmenopausal women with vasomotor symptoms	Not reported	6 × 20-30min session, over 8 weeks	2–3 h in 8 weeks	2 female coaches with a master’s degree (1 social worker, 1 psychologist)
Mindfulness based interventions (*n* = 6)									
Carmody et al 2011 [8]	Body scan, sitting meditation, mindful stretching, mindfulness of breath, thoughts & emotions, Mindfulness in everyday life	Group	Facilitated	F-2-F	No	No	8 × 2.5 h, weekly + full day + homework 45min/day	20 h + full day	Mindfulness instructor
Garcia et al 2018 [20]	Relaxation and Mindfulness exercises inc. Body scan & breathing	Individual	Facilitated	F-2-F	No	Not reported	8 × 0.5 h, weekly + homework	4 h + 24-40min/day for 8 weeks	Mindfulness instructor
Gordon et al 2021 [23]	Psycho-ed on relaxation & mind–body link. Meditation & yoga practice, problem solving	Group	Facilitated	F-2-F	No	No	8 × 2.5 h, weekly + 1 × 7h silent retreat + Homework 45min/day	27h	Mindfulness instructor & master’s level psychologist
Mojtehedi et al 2022 [46]	Meditation, body scan, breathing exercises, present moment and sensory awareness. Accept and allow, cognitive restructuring and self-care	Group	Facilitated	F-2-F	Effects of Mindfulness-Based Intervention on sexual function, anxiety and depression in postmenopausal women	No	8 sessions, weekly. Length not stated	Not reported	MSc student of counselling in midwifery, qualified in MBI
van Driel et al 2019a, b [61, 62]	Mindful eating & walking, body scan, mindfulness of breath, body, thoughts & sounds, yoga, pleasant experiences journal, stress triggers, communication skills, self-care plan and letter to self	Group	Facilitated	F-2-F	No	No	8 × 2.5 h, weekly + 1 × 4h silent retreat + homework 30- 45min/6 days	24 h	Three certified and experienced MBSR trainers
Wong et al 2018 [64]	Stress-reduction skills which included sitting and walking meditation, yoga and body scan	Group	Facilitated	F-2-F	No	No	8 × 2.5 h session, weekly + 45 min/day for homework	20 h + homework	Two clinical psychologists and one nurse
Other interventions (*n* = 8)									
Anderson et al 2015 [2]	Assessed menopausal symptoms, physical, health & lifestyle factors, health education based on assessment to improve positive, health behaviours and goal setting	Individual	Both	F-2-F & Self- help	Lifestyle changes to manage menopausal symptoms in women with breast cancer	Yes	Weekly module for 12 weeks. Monthly telephone, email and letters consult	Hours not specified	Healthcare professional/ Nurse
Augoulea et al 2021 [4]	Stress management. Education on exercise and diet, cognitive restructuring, progressive neuromuscular relaxation, and guided visualization	Group	Facilitated	F-2-F	No	No	8 sessions, weekly. Length not specified	Not specified	Not stated
Fenlon (1999) [17]	Relaxation therapy focusing on hot flushes for women with breast cancer	Individual	Both	F-2-F & Self- help	Relaxation therapy for hot flushes in women with breast cancer	Yes	2 sessions, weekly followed by daily self-practice for 1 month	2 h + self-practice	Occupational therapist
Fenlon et al 2008 [18]	Psycho-ed on stress management, deep breathing, muscle relaxation, guided imagery	Individual	Both	F-2-F & Self- help	No	No	1 × 1 h + homework 20min/day for a month	1 h in 1 month	Occupational therapist
Hsiu-Chin et al 2015 [32]	Introduction to sleep disturbances, improvement strategies, stress-reliving exercises, breathing exercises, PMRT and sleep hygiene	Group	Facilitated	F-2-F & Telephone	Sleep quality intervention for menopausal women with sleep disturbances	No	4 × 2 h, over 8 weeks	8 h + 30 min/day of homework	Researcher and stress-relief expert
Jokar et al 2016 [37]	Theoretical and practical training sessions on Benson relation technique	Group	Facilitated	F-2-F & Telephone	Relaxation therapy for insomnia in menopausal women	No	2 × 2 h, weekly followed by daily 30-min home practice for 30 days	4 h of face-to-face training + 15 h of home practice for 30 days	Trained instructors
Lindh-Åstrand & Nedstrand (2013) [39]	Psycho-ed on menopause & applied relaxation for mgmt. of HFNS. Relaxation techniques	Group	Facilitated	F-2-F	Applied relaxation on vasomotor symptoms in postmenopausal women	No	10 × 1 h, weekly	10 h	Therapist/researcher
Monfaredi et al 2022 [47]	Psycho-ed, mindfulness training, acceptance techniques, cognitive diffusion, values-based goal setting and homework	Group	Facilitated	F-2-F	No	No	8 × 1–1.5 h, weekly	8–12 h + homework	First Author

*CR* Cognitive restructuring, *CBT* Cognitive behavioural therapy, *F-2-F* Face-to-face, *HFNS* Hot Flush/Night Sweats, *MB*I Mindfulness-based intervention, *MBSR* Mindfulness-Based Stress Reduction, *PMRT* Progressive Muscle Relaxation Therapy

### Quality assessment

The risk of bias for included studies was assessed using the Cochrane Collaboration’s Risk of Bias 2.0 tool (RoB 2) for randomised trials [59]. One reviewer (KR) independently assessed the quality of included studies, with a second reviewer (MM) assessing a random 35% of papers. Disagreements were resolved through discussion.

### Data analysis and synthesis

Intervention characteristics were summarised (Table 2) to support the narrative synthesis, with retention used as a marker of feasibility. Medians and ranges were converted to means and standard deviations where possible. Studies without sufficient data were narratively synthesised, some contributing to the meta-analysis for certain symptoms but others not due to incomplete reporting.

The meta-analysis was conducted in Stata 17.0 [58] using post-test data to align with previous reviews [11, 61] and maximise available data while reducing heterogeneity [31]. Effect sizes were reported as Hedges’ g with 95% confidence intervals. Following [12] thresholds of 0.2, 0.5, and 0.8 were used to classify small, medium, and large effects.

For trials with multiple psychosocial arms, interventions were combined to assess overall effectiveness. Where necessary, outcomes were transformed to ensure consistency in scale and direction. Subgroup analyses were conducted by treatment type (CBT, mindfulness, other), menopause type (natural, treatment-induced, both/not specified), and follow-up duration. Follow-ups were classified as short-term (≤ 12 weeks) or medium-term (≥ 12 weeks); if multiple short-term follow-ups were reported, the longest was used for post-test analysis.

A random-effects model was used to account for between-study heterogeneity given the diversity of interventions [31]. Forest plots were used to display the study estimates and heterogeneity was assessed with the I^2^ statistic, with < 30% indicating low, up to 50% moderate, 50–75% substantial, and > 75% considerable heterogeneity [31]. Publication bias was evaluated using funnel plots when ≥ 10 studies were available [31, 44].

A sensitivity analysis was conducted using change scores (pre–post differences) instead of post-test scores, with the same sample sizes. Effect sizes were calculated using [49] bias-corrected method, with a conservative pre-test post-test correlation of 0.7 imputed in line with previous analyses. [57].

## Results

A PRISMA flowchart (Fig. 1) summarises the screening process. Of 2,980 records, 28 studies met inclusion criteria; 24 contributed outcome data for the meta-analysis and four were narratively synthesised. Studies were published between 1999 and 2024.

### Study and participant characteristics

Details are shown in Table 1. Control conditions included waitlist, active comparators, treatment-as-usual, and placebo. Most trials (*n* = 23) had two arms, with post-intervention assessments typically at 3–12 weeks. Across 2,887 participants (*n* = 19–254 per study), mean ages ranged 47–58 years. Eleven studies recruited women with natural-onset menopause, nine recruited women with treatment-induced menopause, four included both, and four did not specify menopause type. Sociodemographic characteristics were reported inconsistently across studies, although most samples were predominantly White. The studies were conducted in Australia, Brazil, Canada, China, Egypt, Greece, Iran, the Netherlands, Saudi Arabia, Sweden, the UK, and the USA.

### Intervention characteristics

Table 2 summarises all 28 interventions included in this review. 14 were CBT-based, six were mindfulness-based interventions and eight were captured under other psychosocial approaches. Sessions were typically weekly, lasting 4–20 weeks with contact hours ranging from 1–27 h. Delivery varied, with most interventions delivered face-to-face in groups, and others delivered individually, online, or by telephone. 18 interventions specifically targeted menopause with only seven interventions tailored for individual needs. Follow-up duration ranged from three weeks to one year.

### Risk of bias

**Fig. 2:**
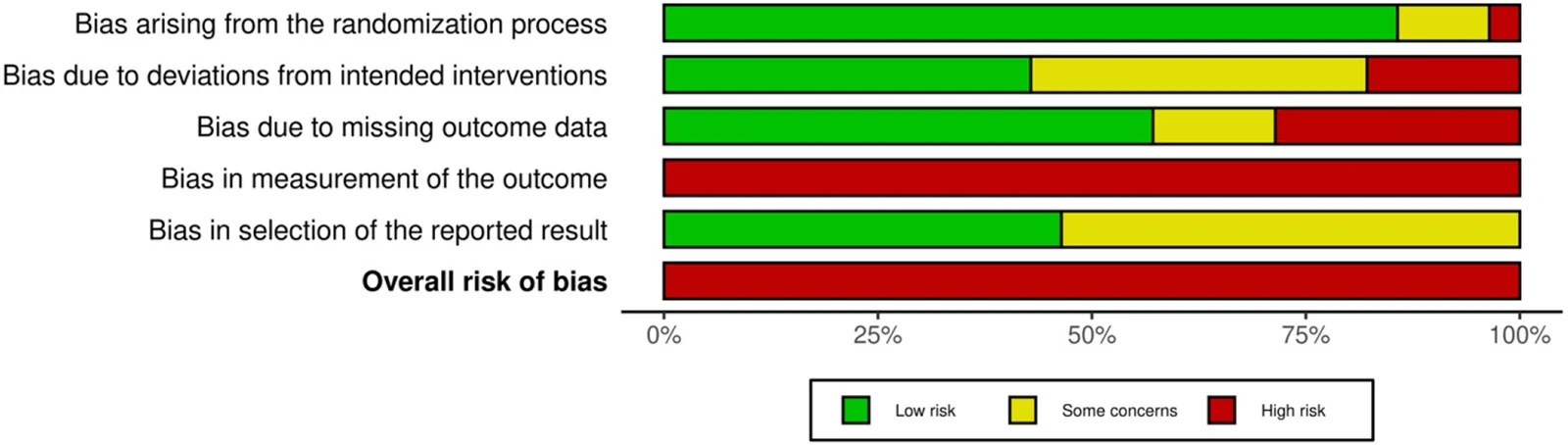
Summary of Bias Domains by Percentage using Risk of Bias 2 Tool

**Fig. 3:**
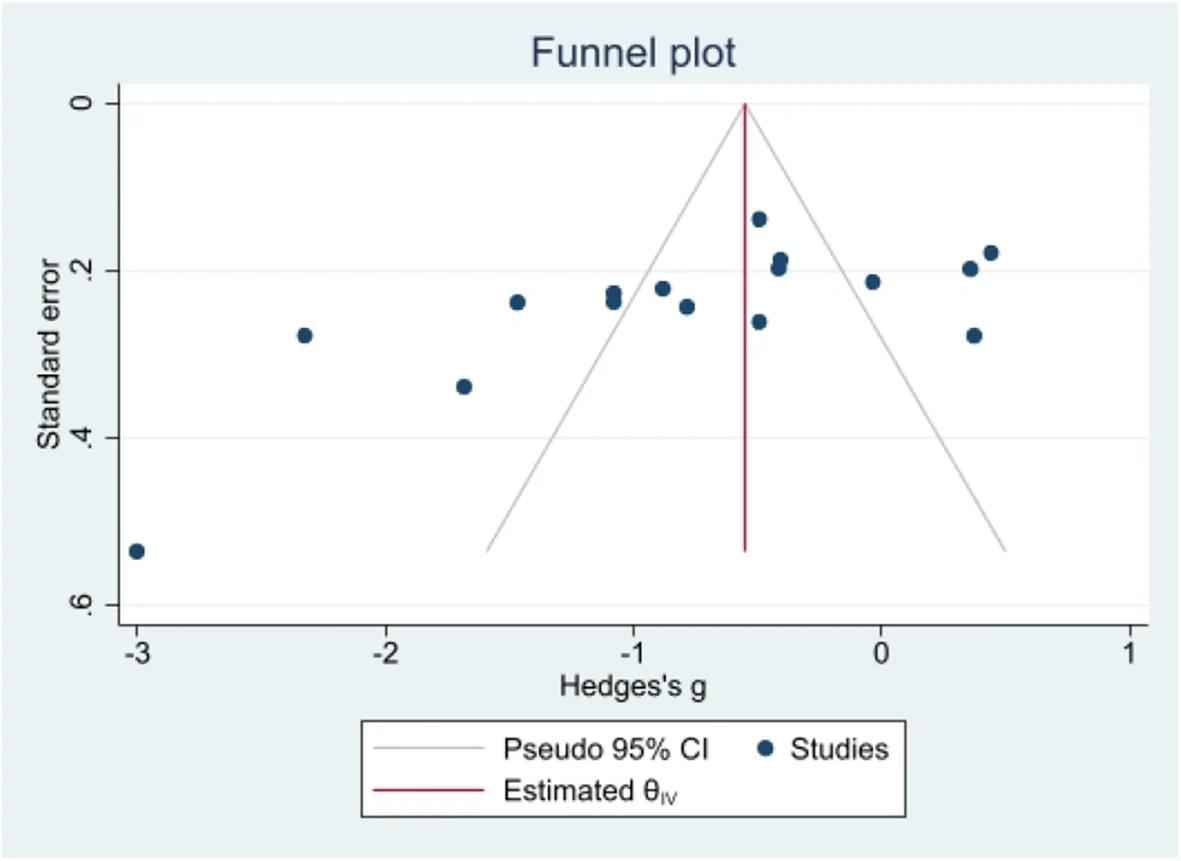
Funnel Plot for Sleep Quality Outcome

All studies were rated as high risk of bias (Appendix 2), primarily due to reliance on self-reported symptom measures, lack of participant blinding and not explicitly stating a pre-specified analysis plan. The distribution of risk-of-bias judgments by percentage within each bias domain is shown in Fig. 2. Funnel plots were examined, and these indicated mild asymmetry. This suggests there may be minor publication bias, where smaller studies may be underrepresented in the literature (Fig. 3). This could also reflect heterogeneity or methodological differences across studies.

### Meta-analytic findings

A meta-analysis was performed pooling data for the outcomes of physical symptoms including VMS (bothersomeness, frequency and severity), sleep (quality and insomnia), sexual functioning, fatigue, pain and urogenital symptoms. VMS bothersomeness was defined as the mean of three 10-point items assessing perceived problem, distress, and interference with daily routine. For clarity, short-term, medium-term, and long-term denote follow-up timing, whereas small, medium, and large refer to the magnitude of effects as indicated by Hedges’ g.

### Vasomotor Symptoms (VMS)

#### HF/NS Bothersomeness

**Fig. 4:**
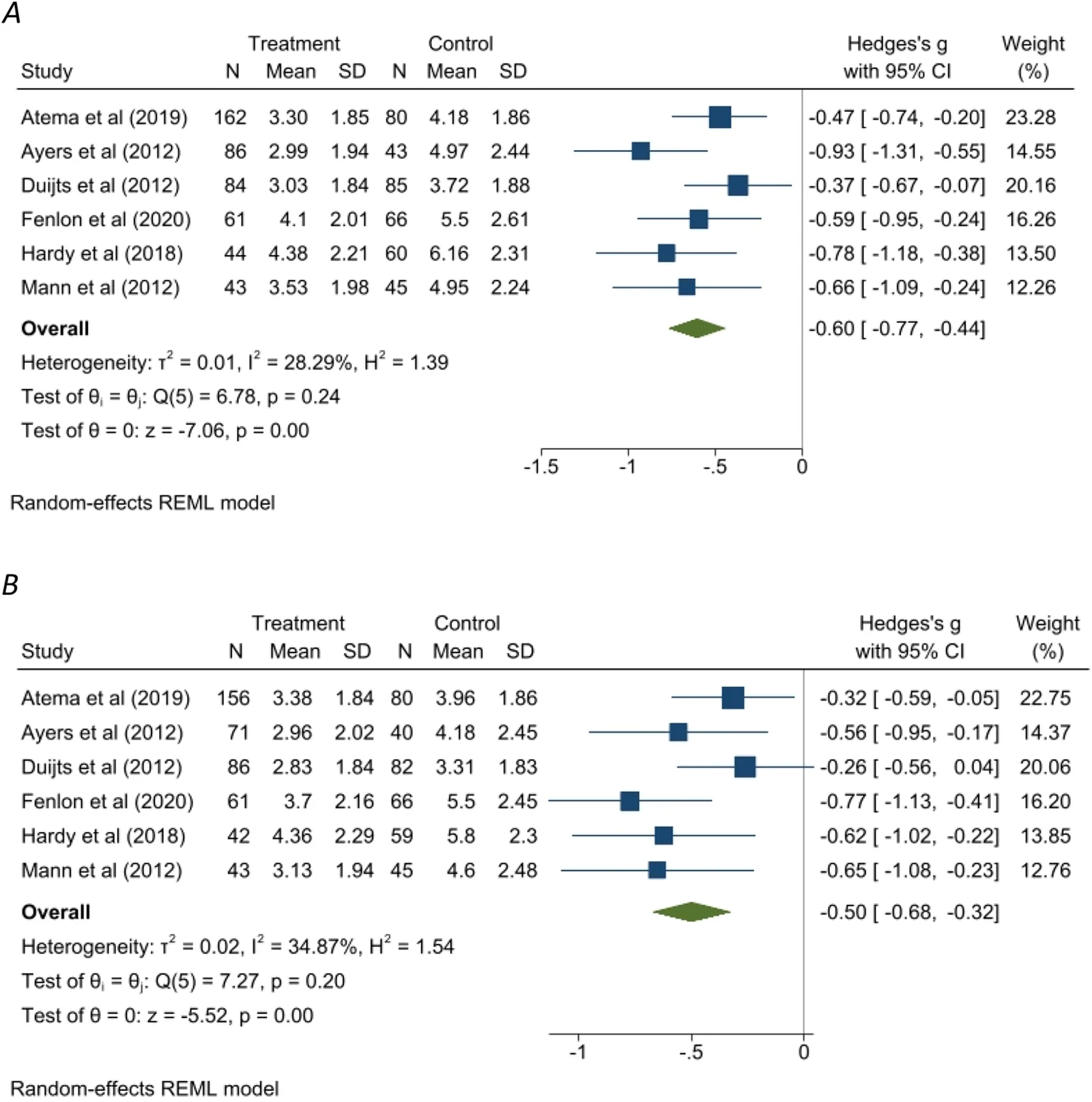
**A** Forest Plot of Meta-analysed Short-term HF/NS Bothersomeness outcome. **B** Forest Plot of Meta-analysed Medium-term HF/NS Bothersomeness outcome

Meta-analysis of six studies (*n* = 480; Fig. 4a) showed a medium effect of CBT-based psychosocial interventions on short-term HF/NS bothersomeness (g = −0.60, 95% CI [−0.77, −0.44]) with low, heterogeneity (I^2^ = 28.29%). A similar medium effect was observed at medium-term follow up across the same studies (*n* = 459; Fig. 4b) (g = −0.50, 95% CI [−0.68, −0.31]) with moderate, heterogeneity (I^2^ = 34.87%). Subgroup analyses by intervention type were not conducted as all included interventions were CBT-based. Sensitivity analyses produced results consistent with the main findings (Appendix 3.1).

### HF/NS frequency

**Fig. 5:**
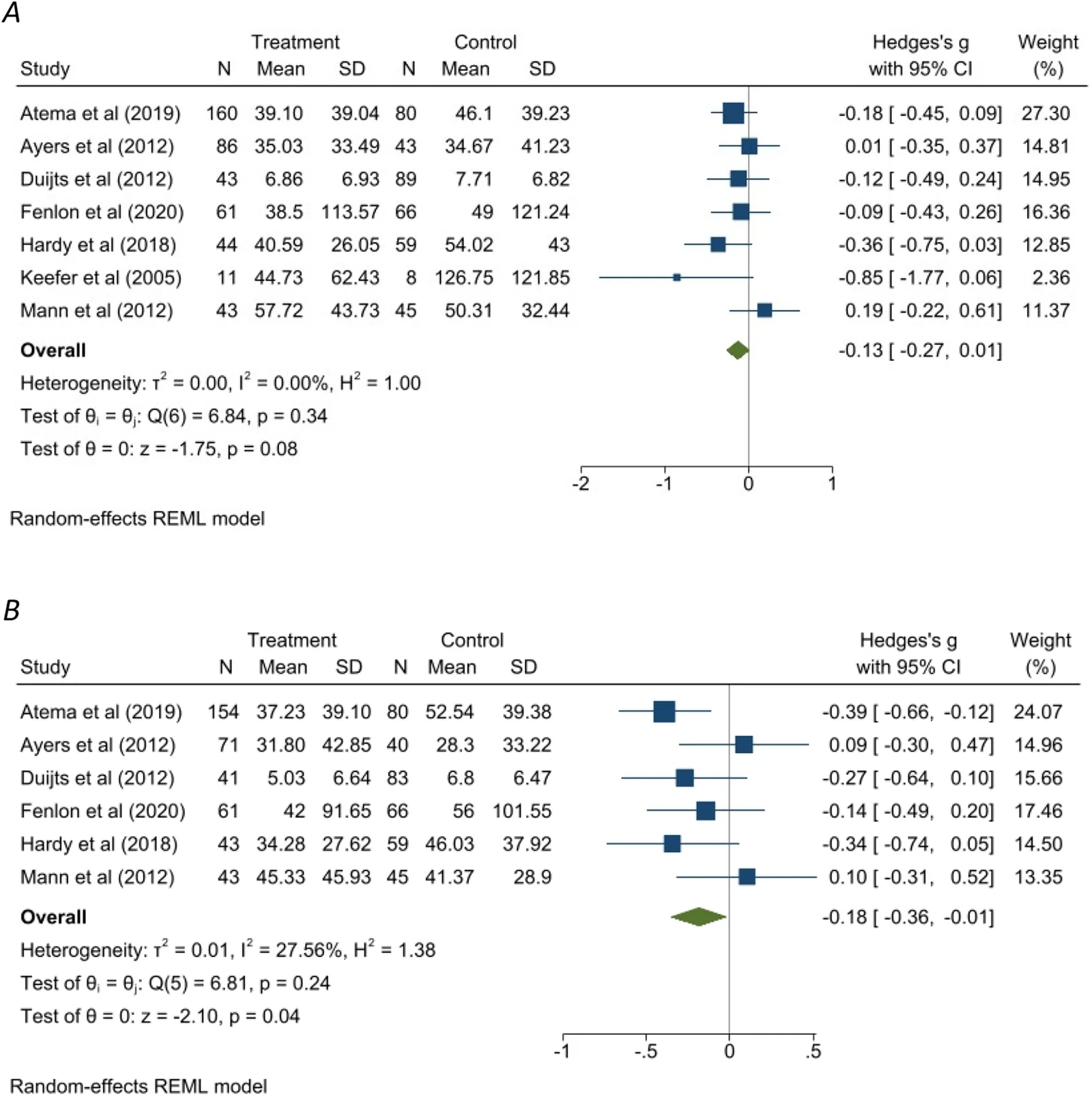
**A** Forest Plot of Meta-analysed Short-term HF/NS Frequency Outcome. **B** Forest Plot of Meta-analysed Medium-term HF/NS Frequency Outcome

Meta-analysis of seven studies (*n* = 448; Fig. 5a) showed no effect of psychosocial interventions on short-term HF/NS frequency (g = −0.13, 95% CI [−0.27, 0.01]; I^2^ = 0.0%).

No significant difference was observed between CBT and other interventions at short-term follow-up (Appendix 3.2). Meta-analysis of six studies (*n* = 413; Fig. 5b) showed a small effect of psychosocial interventions on medium-term HF/NS frequency (g = −0.18, 95% CI [−0.36, −0.01]) with low heterogeneity (I^2^ = 27.56%). Since only CBT interventions had medium-term follow-up, no subgroup analysis was conducted.

Sensitivity analyses using change scores indicated small effects on HF/NS frequency at both short- and medium-term follow-up (Appendix 3.2).

### Vasomotor severity

**Fig. 6:**
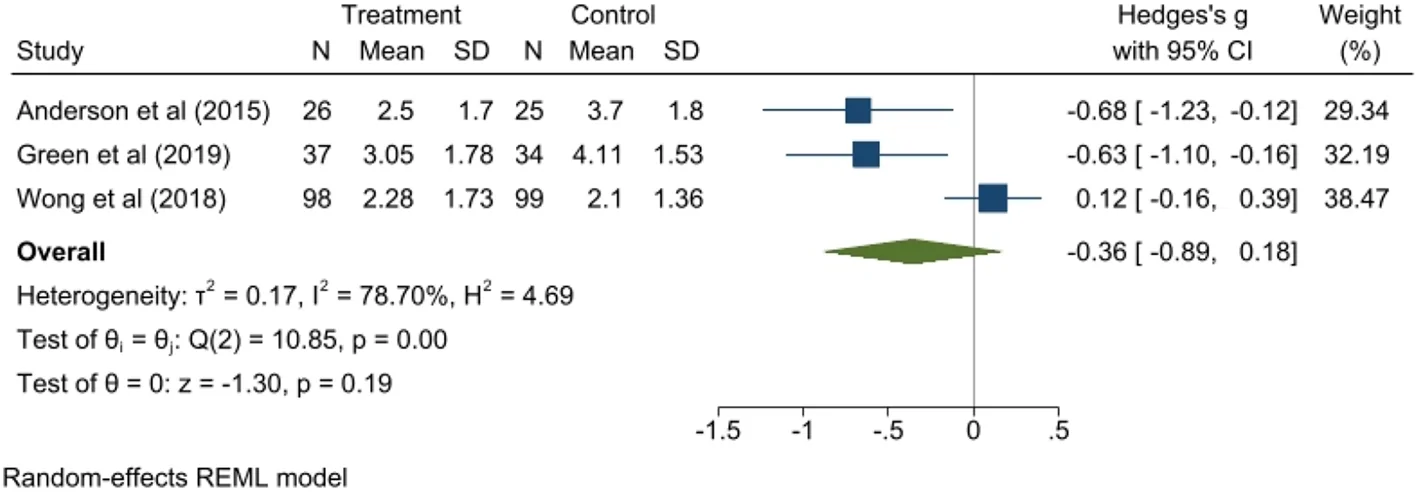
Forest plot of Meta-analysed short-term HF/NS Bothersomeness outcome

Meta-analysis of three studies (*n* = 161; Fig. 6) found no effect of psychosocial interventions on vasomotor severity (g = −0.36, 95% CI [−0.89, 0.18]) with considerable heterogeneity (I^2^ = 78.7%). Subgroup analyses were not conducted as each study evaluated a different intervention. Sensitivity analysis using change scores (Appendix 3.3) found a small effect of psychosocial interventions on vasomotor severity with no heterogeneity..

### Sleep

#### Sleep Quality

**Fig. 7:**
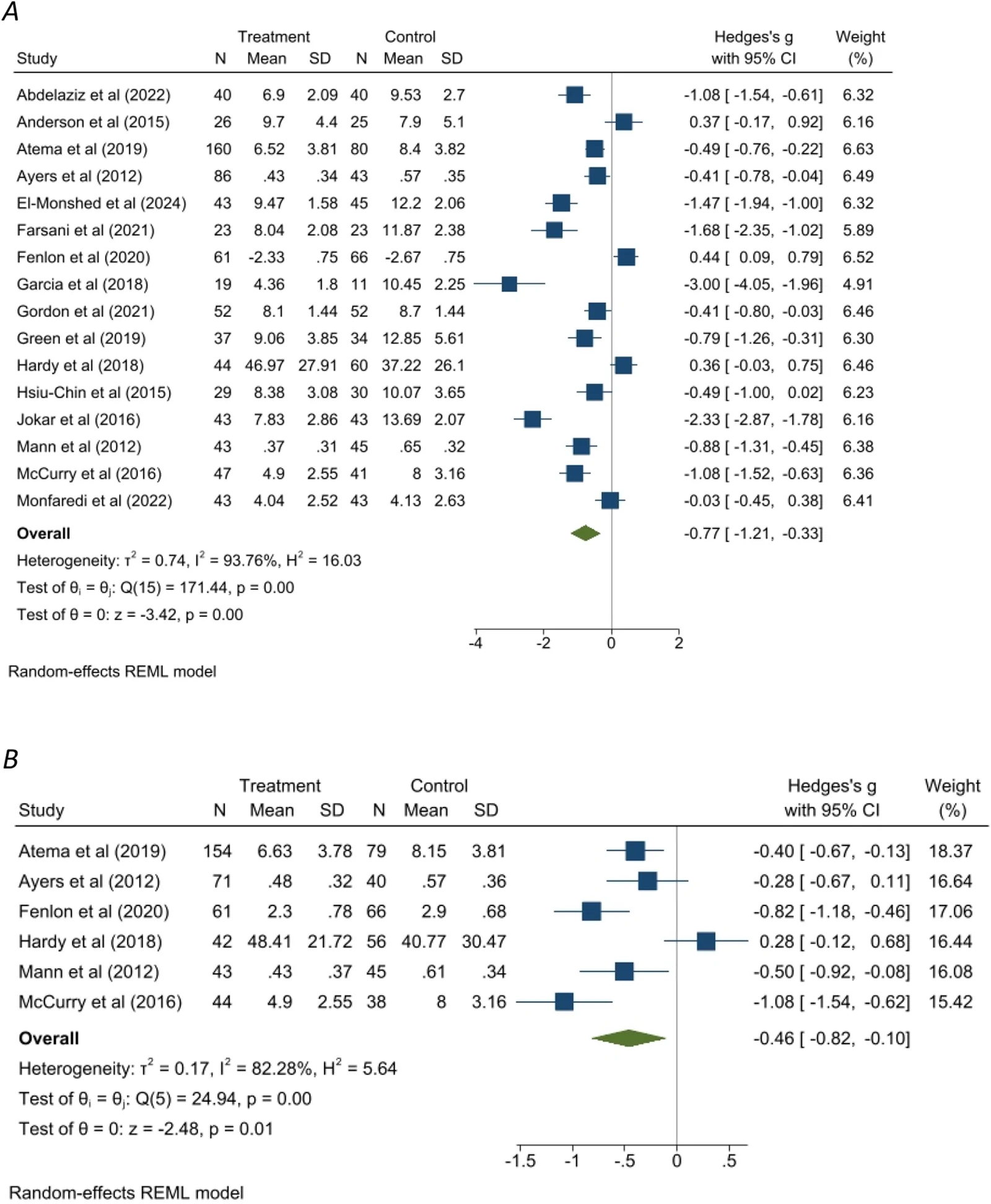
**A** Forest plot of Meta-analysed Short-term Sleep Quality Outcome. **B** Forest Plot of Meta-Analysed Medium-term Sleep Quality Outcome

Meta-analysis of 16 studies (*n* = 792; Fig. 7a) showed a medium effect of psychosocial interventions on short-term sleep quality (g = −0.77, 95% CI [−1.21, −0.33]), with considerable heterogeneity (I^2^ = 93.76%). A subsequent meta-analysis of six studies (*n* = 415; 7b) showed a medium effect of psychosocial interventions on medium-term sleep quality (g = −0.46, 95% CI [−0.82, −0.10]) with considerable but non-significant heterogeneity (I^2^ = 82.28%). All medium-term studies evaluated CBT. Sensitivity analyses indicated a large short-term improvement in sleep quality but no medium-term effect (Appendices 3.4–3.5).

**Fig. 8:**
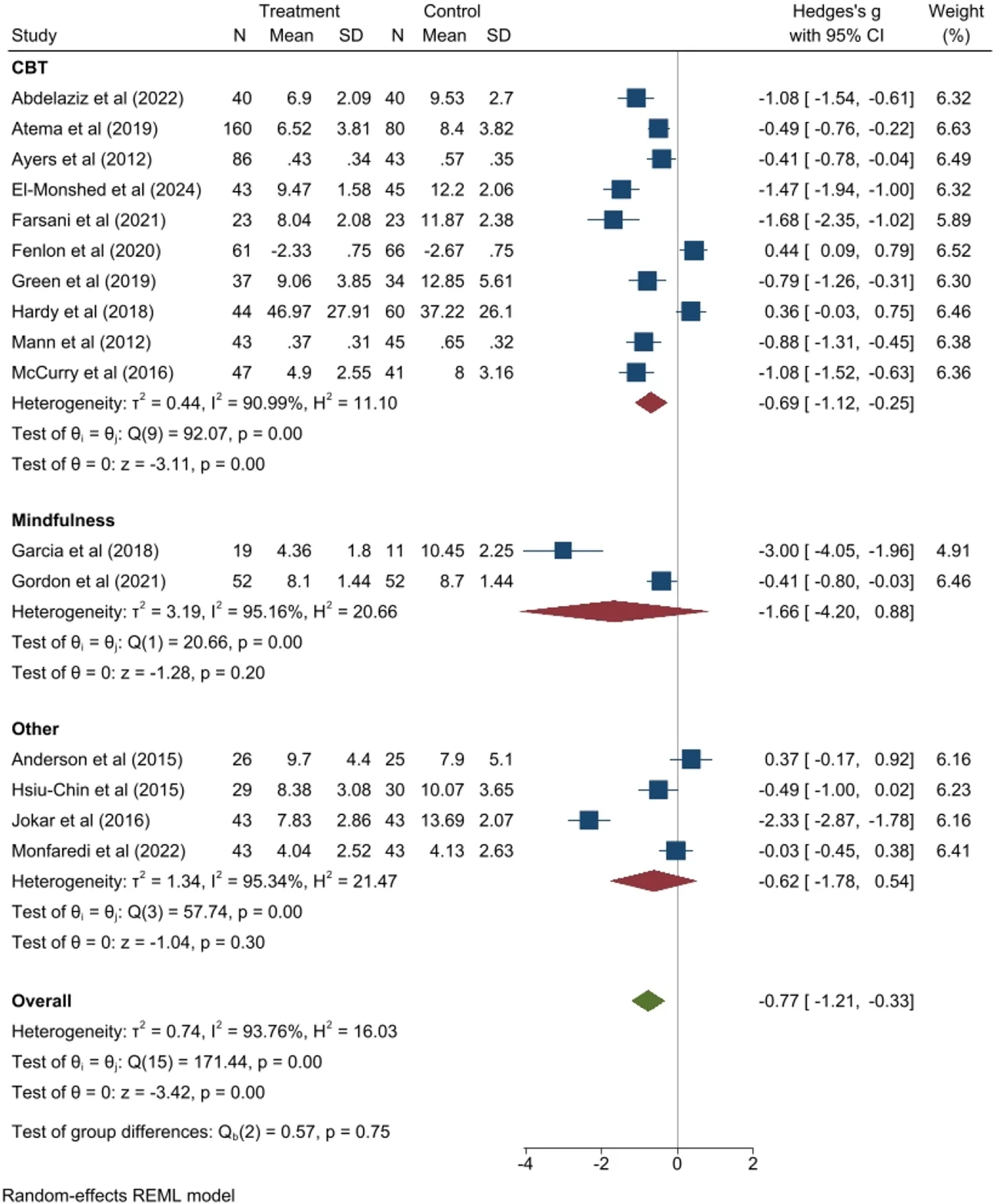
Forest plot of short-term effects of psychosocial interventions on sleep quality, stratified by intervention type

Subgroup analysis of short-term sleep quality by intervention type (Fig. 8) found medium effects for CBT (g = −0.69, 95% CI [−1.12, −0.25]; I^2^ = 90.99%) but no effect for mindfulness (g = −1.66, 95% CI [−4.20, 0.88]; I^2^ = 95.16%) or other interventions (g = −0.62, 95% CI [−1.78, 0.54]; I^2^ = 95.3%). No differences in effect size were observed between treatment types (*p* = 0.75). Subgroup analysis by menopause type also found no significant differences between groups (*p* = 0.08).

### Insomnia

**Fig. 9:**
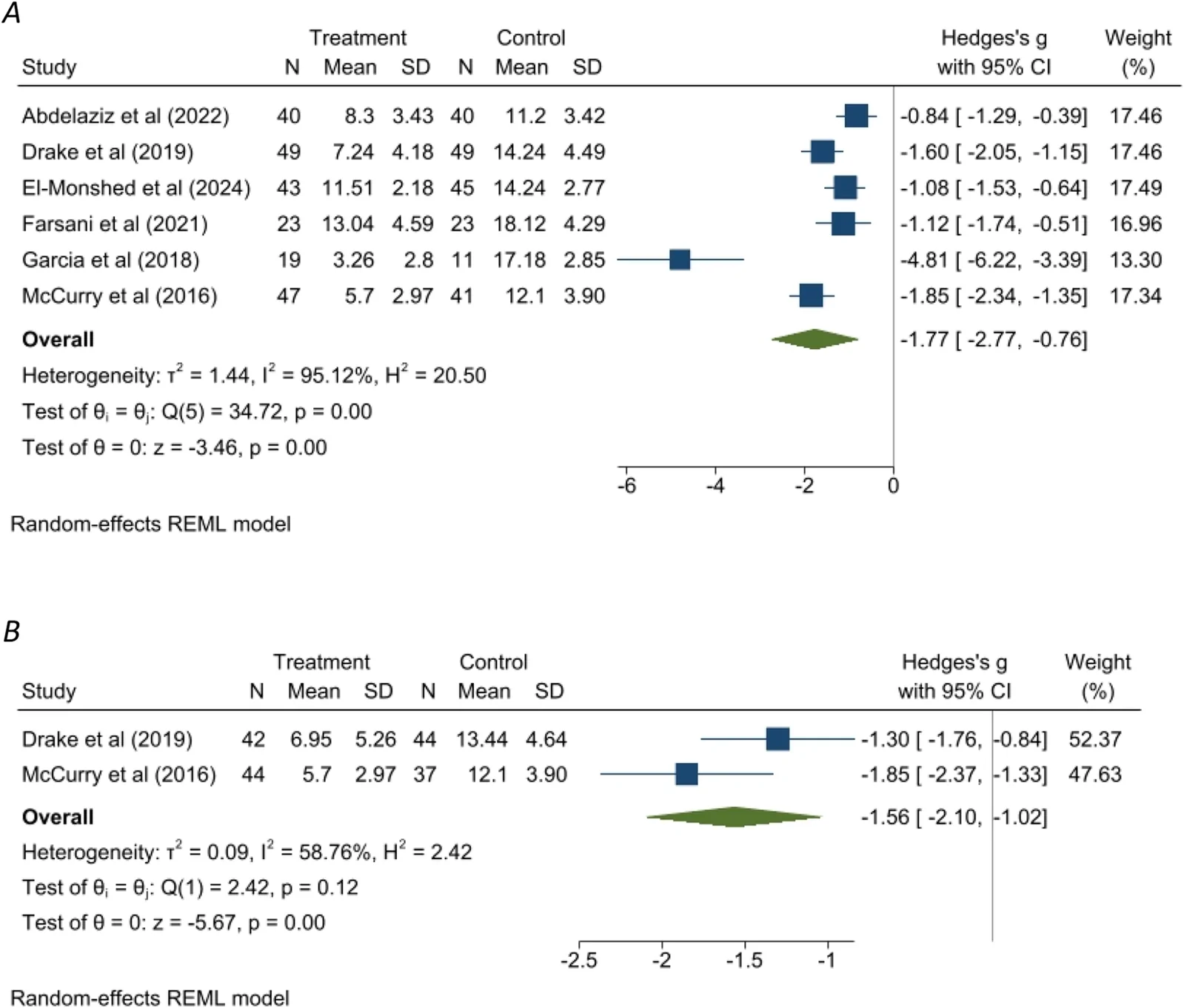
**A**: Forest Plot of Meta-analysed Short-term Insomnia Outcome. **B** Forest Plot of Meta-analysed Medium-term Insomnia Outcome

Meta-analysis of six studies (*n* = 221; Fig. 9a) showed a large effect of psychosocial interventions on improving short-term insomnia severity (g = −1.77, 95% CI [−2.77, −0.76]), with significant heterogeneity (I^2^ = 95.12%). A subsequent meta-analysis of two studies (*n* = 86; 9b) showed a large effect of psychosocial interventions on improving medium-term insomnia severity (g = −1.56 = X, 95% CI [−2.10, −1.02]), with substantial significant heterogeneity (I^2^ = 58.76%). All studies measuring medium-term effects were CBT.

**Fig. 10:**
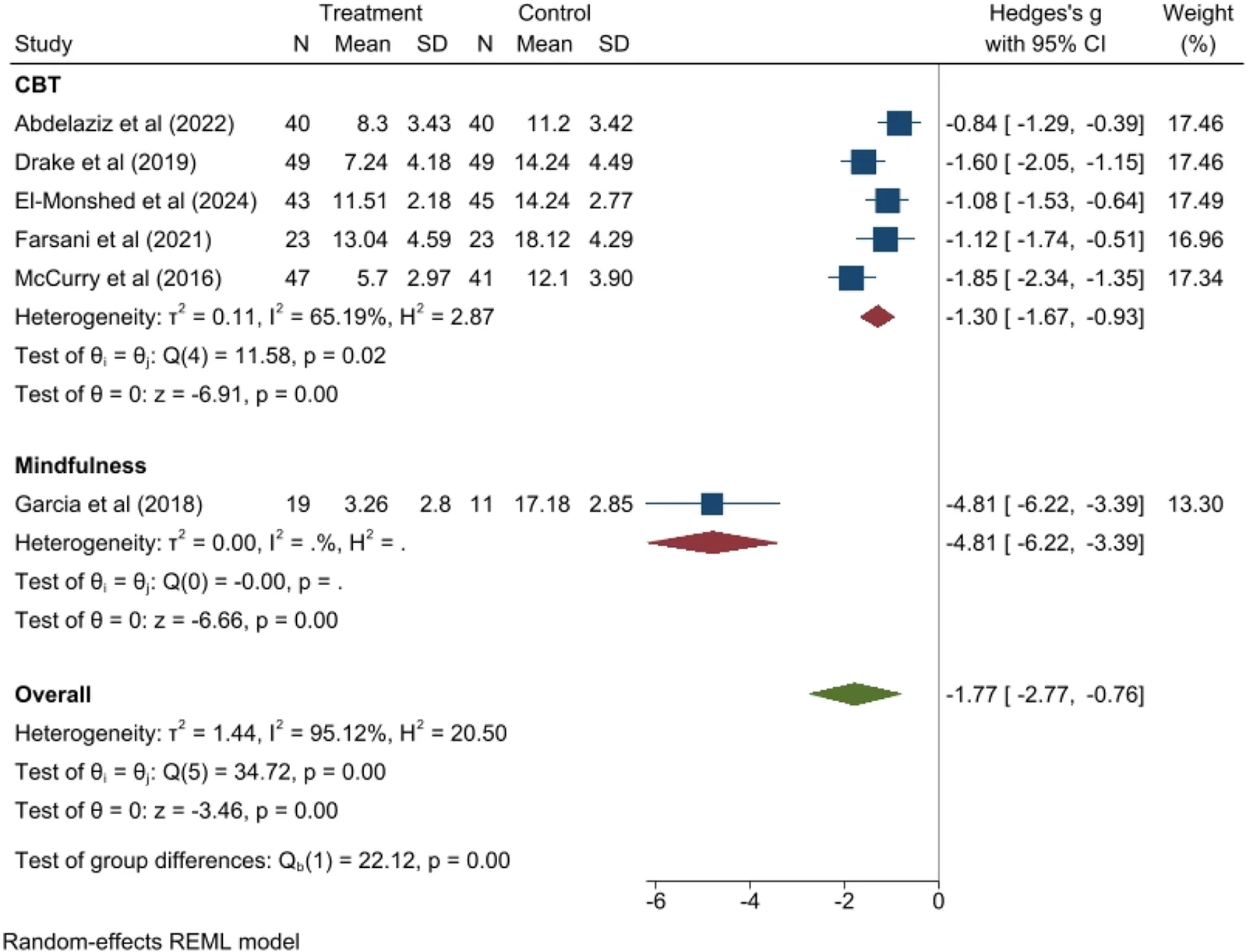
Forest plot of short-term effects of psychosocial interventions on insomnia, stratified by intervention type

A subgroup analysis was conducted for intervention type (Fig. 10). CBT interventions produced a large improvement in short-term quality of sleep (g = −1.30, 95% CI [−1.67, −0.93]), with substantial heterogeneity (I^2^ = 65.19%). The effect of mindfulness was also large (g = −4.81, 95% CI [−6.22, −3.39]) but was based on only 1 study. There was a statistically significant difference between CBT and mindfulness (*p* < 0.01) but no difference in effect between menopause type (*p* = 0.47). Sensitivity analyses indicated larger short-term and medium-term improvements in insomnia severity, with results consistent across subgroups (Appendices 3.6–3.7).

### Sexual Functioning

**Fig. 11:**
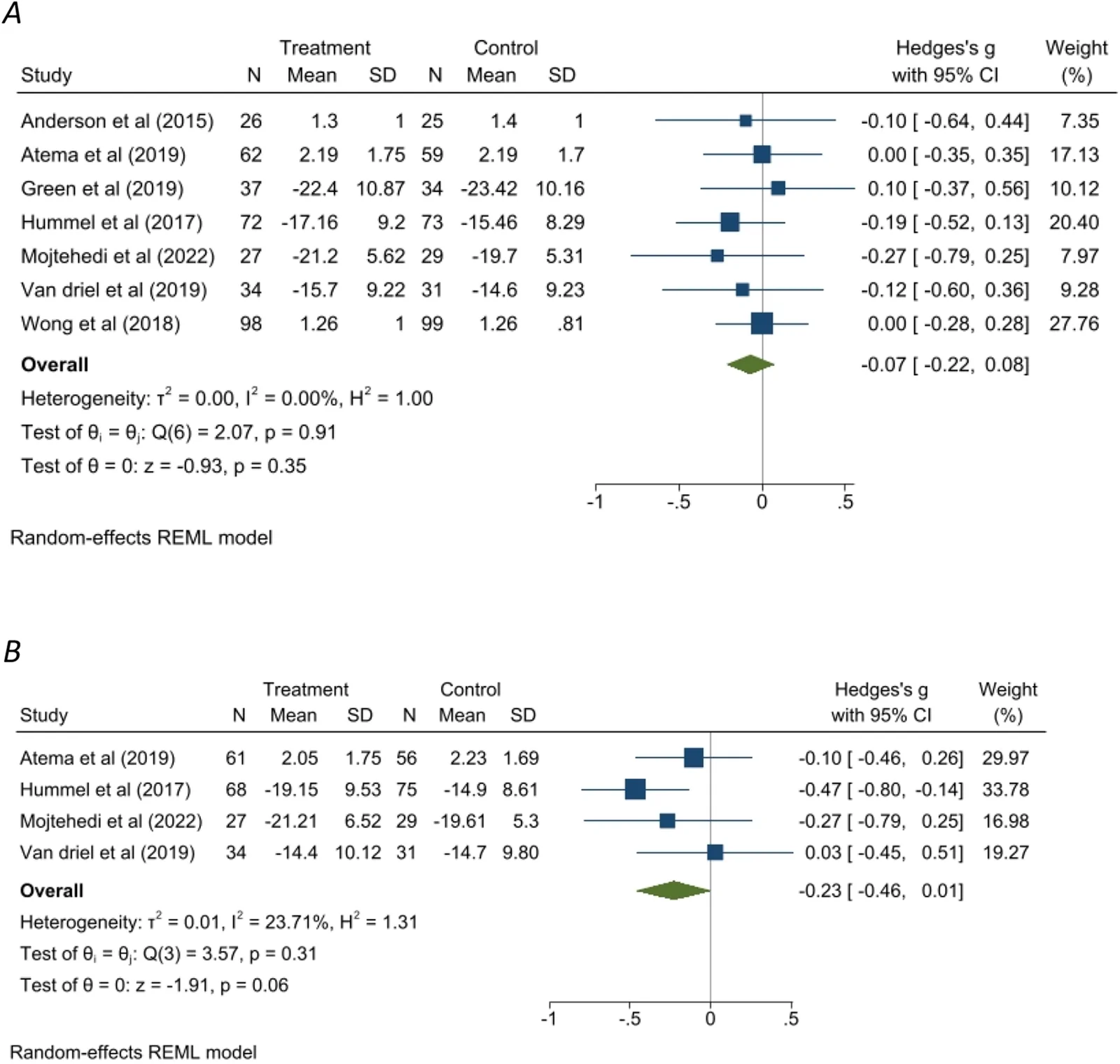
**A** Forest Plot of Meta-analysed Short-term Sexual Functioning Outcome. **B** Forest Plot of Meta-analysed Medium-term Sexual Function Outcome

Meta-analysis of seven studies (*n* = 356; Fig. 11a) showed no effect of psychosocial interventions on improving short-term sexual functioning (g = −0.07, 95% CI [−0.22, 0.08]), with no heterogeneity (I^2^ = 0.00%). Similarly, a subsequent meta-analysis of four studies (*n* = 190; Fig. 11b) showed no effect of psychosocial interventions on improving medium-term sexual functioning (g = −0.23, 95% CI [−0.46, 0.01]), with low heterogeneity (I^2^ = 23.71%).

**Fig. 12:**
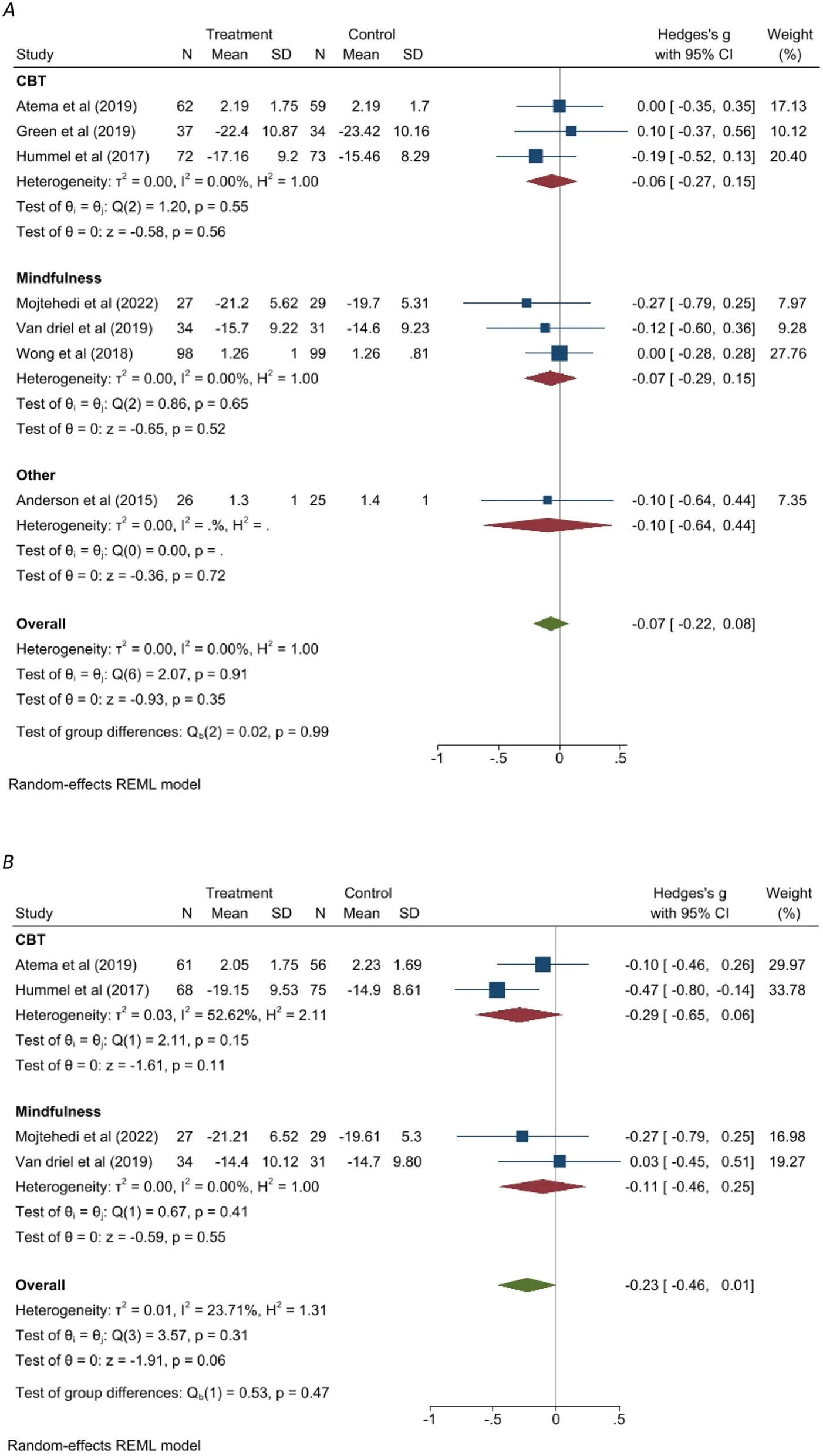
**A** Forest plot of short-term effects of psychosocial interventions on sexual functioning, stratified by intervention type. **B** Forest plot of medium term effects of psychosocial interventions on sexual functioning, stratified by intervention type

Subgroup analysis by intervention type (Fig. 12a) found no short-term effect for sexual functioning following CBT or mindfulness respectively (g = −0.06, 95% [CI −0.27, 0.15]; g = −0.07, 95% CI [−0.29, 0.15]), with no heterogeneity (I^2^ = 0.00%). Similarly, subgroup analysis by intervention type for medium term follow up (Fig. 12b) also found no significant difference between CBT and Mindfulness (p = 0.47). Sensitivity and subgroup analyses yielded results consistent with the main findings (Appendix 3.8; 3.9).

### Fatigue

**Fig. 13:**
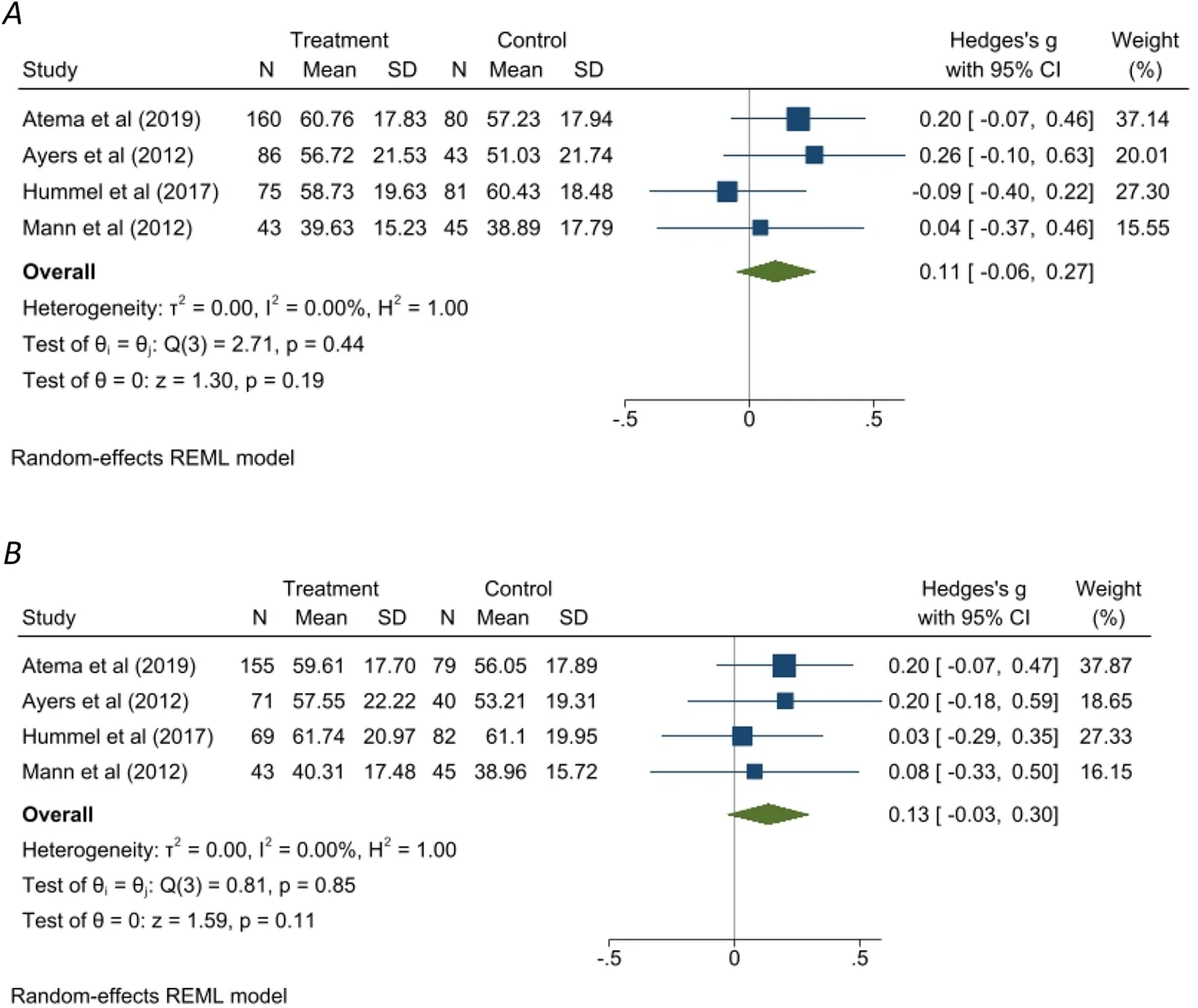
**A** Forest Plot of Meta-analysed Short-term Fatigue Outcomes. **B** Forest Plot of Meta-analysed Medium-term Fatigue Outcomes

Meta-analysis of four studies (*n* = 364; Fig. 13a) found no effect of psychosocial interventions on short-term fatigue (g = 0.11, 95% CI [−0.06, 0.27]). Similarly, a meta-analysis of four studies at medium-term follow up (*n* = 338; Fig. 13b) found no effect (g = 0.13, 95% CI [−0.03, 0.30]). No heterogeneity was observed at either time point (I^2^ = 0.00%). Subgroup analysis was not conducted as all interventions were CBT-based. Sensitivity analyses showed similar results, with a small but significant medium-term improvement in fatigue and no heterogeneity (Appendix 3.10).

### Pain

**Fig. 14:**
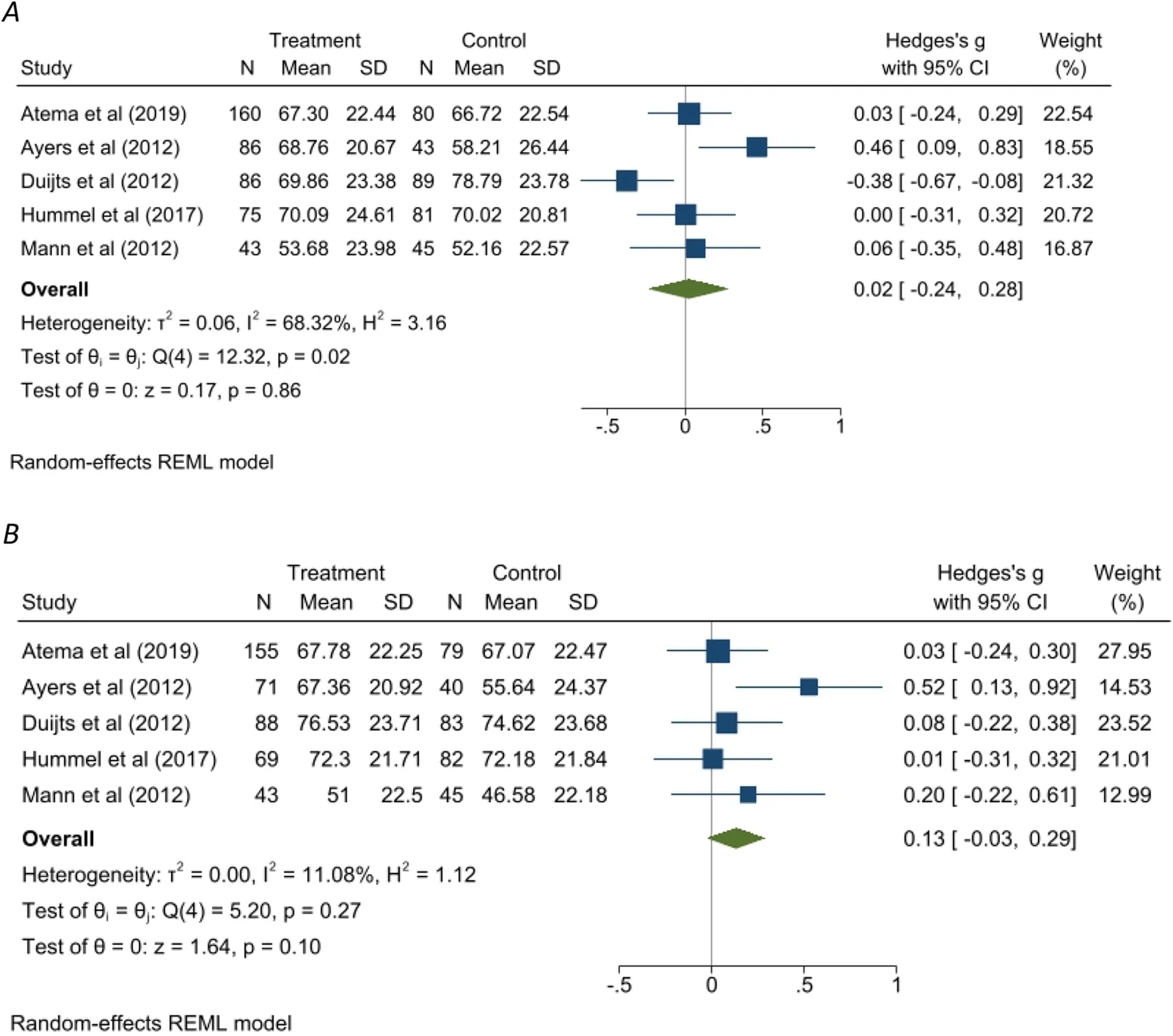
**A** Forest Plot of Meta-analysed Short term Pain Outcomes. **B** Forest Plot of Meta-analysed Medium-term Pain Outcomes

Meta-analysis of five studies (*n* = 450; Fig. 14a) showed no effect of psychosocial interventions on improving short-term pain (g = 0.02, 95% CI [−0.24, 0.28]) with significant and substantial heterogeneity (I^2^ = 68.32%). Similarly, a meta-analysis of the same five studies (n = 426; Fig. 14b) showed no effect of psychosocial interventions on improving medium-term pain (g = 0.13, 95% CI [−0.03, 0.29]), with significant and low heterogeneity (I^2^ = 11.08%). Subgroup analysis was not conducted as all interventions were CBT-based.

Sensitivity analyses showed similar results, with a small medium-term improvement in pain and minimal heterogeneity (Appendix 3.11).

### Urogenital

**Fig. 15:**
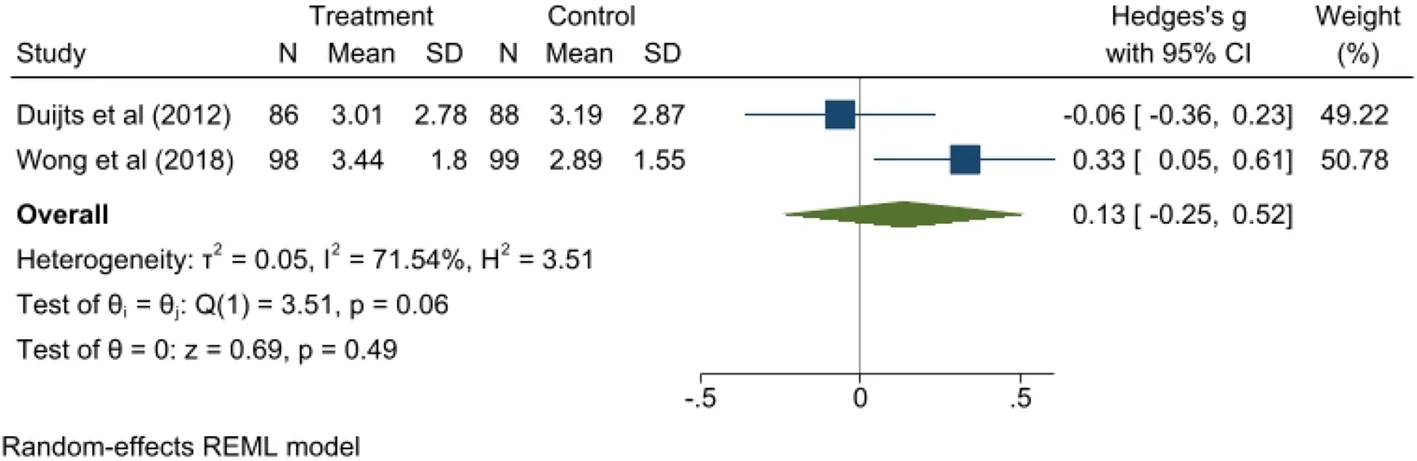
Forest Plot of Meta-analysed Short-term Urogenital Outcomes

Meta-analysis of two studies (*n* = 184; Fig. 15) showed no effect of psychosocial interventions on short-term urogenital symptoms (g = 0.13, 95% CI [−0.25, 0.52]) with considerable and non-significant heterogeneity (I^2^ = 71.54%). Sensitivity analysis (Appendix 3.12) found no effect.

### Narrative synthesis including additional studies

Four studies were not included in any meta-analysis due to insufficient data [4, 8, 17] Lindh-Astrand & Nedstrand, [39]. In addition, some studies [17, 18, 38] reported problem, distress, and interference factors separately rather than the combined ‘problem rating scale’ now commonly used [36] and therefore could not be included.

### Hot flush/night sweat bothersomeness

Relaxation-based interventions showed mixed results: [18] reported short-term improvements in distress (p = 0.01) but no sustained effects, Lindh-Astrand and Nedstrand [39] found short-term benefits (p < 0.05), though effects were not maintained and [17] found no significant differences. An MBSR study [8] reported no significant differences in bothersomeness compared to control. Keefer and Blachard [38] reported reductions in distress and interference following CBT that approached significance, but not in overall problem ratings. Overall, effects were inconsistent and generally short-lived.

### Hot flush/night sweat frequency

Fenlon [17] conducted a relaxation intervention and found no significant difference in the frequency of hot flushes and night sweats experienced in the relaxation group compared to the control group.

### Vasomotor severity

Augoulea et al. [4] evaluated a stress management intervention; however, outcome data for VMS were reported only graphically, without accompanying p-values or confidence intervals. Visual inspection of the graph suggested no meaningful change in VMS in the intervention group and a slight decrease in the control group.

### Sleep quality

Lindh-Astrand & Nedstrand’s [39] relaxation-based intervention found that medium-term, sleep quality was improved in the intervention group compared to the control group (*P* < 0.01), with changes being clinically meaningful.

### Insomnia

Carmody et al. (2011) [8] evaluated a Mindfulness-Based Stress Reduction (MBSR) intervention, with greater sleep quality in MBSR participants compared to controls (*P* = 0.009). There were no medium-term differences between groups (*P* = 0.766).

### Sexual unctioning

As with vasomotor symptom outcomes described above [4], reported sexual functioning data graphically only. Visual inspection suggested no change in sexual functioning in either the intervention or control group.

### Retention rates

Across studies, retention from randomisation to final follow-up ranged 61–100% (mean ≈ 87%), with three studies not reporting retention data. Average retention was similar across intervention types: CBT (87%), mindfulness (84%), and other psychosocial interventions (89%).

## Discussion

### Vasomotor symptoms (VMS)

Consistent with prior evidence [65], CBT-based psychosocial interventions were associated with reductions in the bothersomeness of VMS at short- and medium-term follow-up, while effects on symptom frequency and severity were smaller and less consistent. This distinction is important, as bothersomeness reflects subjective distress, perceived interference and functional impact, capturing how symptoms are experienced and managed in daily life. These findings support the value of targeting cognitive appraisals, emotional responses, and coping strategies to reduce the perceived impact of VMS (Hunter & Mann, [35]). Psychosocial interventions may therefore be considered either alongside medical management (such as MHT) or as a stand-alone treatment option for bothersome VMS, particularly where non-pharmacological approaches are preferred and/or where individuals are not eligible for MHT, such as some breast cancer patients. Findings for non-CBT approaches, including relaxation and mindfulness-based interventions, were mixed, likely reflecting heterogeneity in intervention content, dose, and study design.

### Sleep

Psychosocial interventions were effective in improving menopause-related sleep difficulties, showing medium to large effects that persisted at follow-up. However, effect sizes diminished over time, suggesting that booster sessions or ongoing practice may be required to sustain benefits. CBT demonstrated the most consistent evidence of efficacy, showing medium effects in line with previous reviews [9, 52]. Mindfulness-based interventions showed promising short-term effects, although the small number of studies and mixed follow-up findings warrant cautious interpretation. Other modalities, including relaxation and ACT, also demonstrated medium effects. Overall, while CBT remains the most robust intervention for sleep difficulties, alternative psychosocial approaches may also offer benefit. Larger studies with longer follow-up are needed to determine whether these effects are sustained over time.

### Sexual functioning

Overall, psychosocial interventions did not demonstrate benefit for sexual functioning, with no effects observed at short- or medium-term follow-up and no consistent differences by intervention type [61, 65]. One tailored CBT intervention showed a small benefit at medium-term follow-up, suggesting that more symptom-specific and context-sensitive approaches may be required to address sexual functioning effectively [33]. Given the considerable variability in sexual symptoms across menopausal stages and individual experiences, future research should prioritise stage-specific analyses and consider relational and interpersonal factors, such as relationship dynamics and partner support [48].

### Fatigue and pain

Psychosocial interventions, particularly CBT, were associated with small improvements in fatigue and pain at medium-term follow-up but not at short-term follow up. This pattern partially aligns with prior research reporting small improvements in fatigue but no effect on pain [65] and suggests that time may be required for cognitive and behavioural skills to translate into symptom change. Tailoring interventions to menopause-specific fatigue and pain, such as incorporating activity pacing, behavioural activation, and cognitive restructuring, may enhance effectiveness. Evidence from other chronic health conditions supports the use of CBT for fatigue and pain management (Bermpohl et al., [41]; [63]). However, commonly used outcome measures, including SF-36 subscales, may lack sensitivity to the lived experience of fatigue and pain in menopause, highlighting the need for more targeted assessment tools. Non-CBT approaches, such as Acceptance Commitment Therapy and mindfulness-based interventions, remain underexplored for the management of menopausal fatigue and pain, despite evidence of efficacy for these symptoms in other adult populations [40, 43], warranting further investigation.

### Urogenital

Psychosocial interventions did not demonstrate improvements in urogenital symptoms, with considerable heterogeneity observed across studies. These symptoms are likely to have a stronger biological basis, including oestrogen deficiency and tissue atrophy, and may therefore be more responsive to medical management. Interpretation of findings is further limited by methodological issues, as some studies did not assess urogenital symptoms despite targeting them [24], or relied on broad measures that do not adequately isolate urogenital outcomes, such as the MRQ (Hunter & O’Dea, [34]). Nevertheless, urogenital symptoms can have substantial psychosocial consequences [22], and the current evidence base remains limited [60]. Future research is therefore needed to clarify whether psychosocial interventions may play a supportive role alongside medical treatments for urogenital symptoms.

### Limitations

Several included interventions were designed to target specific symptoms or populations (e.g., VMS, insomnia, or menopausal symptoms in breast cancer survivors), whereas some outcomes included in the meta-analysis were secondary measures rather than primary treatment targets. As such, pooled effects for certain symptom domains may underestimate intervention efficacy where those outcomes were not the main focus of treatment. Intervention effectiveness by menopausal stage could not be determined, as many studies did not report or analyse outcomes by stage, despite evidence that symptom presentation and impact vary across the menopausal transition [10]. In addition, no studies assessed headaches or other somatic symptoms independently of psychological outcomes, limiting conclusions regarding psychosocial effects on these symptoms. Only one third-wave intervention, Acceptance Commitment Therapy, met inclusion criteria [47], restricting conclusions about the effectiveness of third wave approaches.

Another limitation of the evidence base relates to the nature of control conditions used across included trials. Control groups varied, with only approximately 60% employing active placebo controls. Given that menopausal symptoms, particularly VMS, are susceptible to placebo effects, this limits the extent to which observed effects can be attributed to the active components of psychosocial interventions. Findings should therefore be interpreted cautiously.

All included studies were rated as high risk of bias, primarily due to reliance on self-reported symptom measures and the lack of participant blinding. While self-report is clinically relevant for subjective outcomes such as distress, sleep quality, and symptom bothersomeness, it remains vulnerable to reporting and expectancy biases. Furthermore, although short- and medium-term outcomes were commonly reported, long-term follow-up data were scarce, limiting conclusions about sustained efficacy. More rigorous trials incorporating active placebo controls, longer follow-up periods and multimethod assessments, including objective or physiological measures where feasible, would strengthen future research.

### Implications for research and practice

Findings highlight the potential of psychosocial interventions to support women during both natural and treatment-induced menopause. Benefits were strongest for CBT, consistent with clinical guidance, including NICE [51], which recommends CBT (general and menopause-specific) as an evidence-based non-pharmacological option for symptoms such as VMS. Mindfulness and other approaches included in this review show promise but require further research. Smaller effects suggest that combining psychosocial and medical interventions, where appropriate, may enhance outcomes. Tailoring interventions to specific symptom domains, such as VMS, insomnia, sexual functioning, and fatigue, could increase effectiveness, while offering a range of approaches is important to meet individual needs and preferences. Future studies should investigate, where feasible, long-term effects and efficacy across different menopause stages.

Retention rates averaged 86.7%, indicating psychosocial interventions for menopause are generally feasible and acceptable. However, access to psychosocial interventions, particularly CBT, may be limited by clinician availability, service costs, and geographic variation in provision, with menopause-specific CBT likely to face additional barriers. CBT and other interventions typically required fewer contact hours than mindfulness, which has implications for delivery, staffing, and costs. Most interventions were group-based, suggesting that peer interaction may enhance effectiveness and efficiency. Scalable digital approaches may help improve accessibility and reach. Overall, the successful delivery of psychosocial support for menopause is likely to depend on careful consideration of both intervention type and delivery format.

## Conclusion

This systematic review and meta-analysis indicates that psychosocial interventions, particularly CBT, can support the management of menopausal symptoms, with the strongest and most consistent effects observed for reductions in VMS bothersomeness and improvements in sleep outcomes. Evidence for other symptoms, including fatigue and pain, was more limited and generally characterised by small effects, while no benefits were observed for sexual functioning or urogenital symptoms. The pattern of findings suggests that psychosocial interventions may primarily influence the subjective experience and impact of symptoms, rather than directly altering symptom frequency or severity. Overall, CBT demonstrated the most robust evidence across symptom domains, while evidence for mindfulness-based, relaxation, and third-wave approaches remains emerging. Future research should prioritise longer-term follow-up, where feasible, alongside further evaluation and refinement of targeted interventions for specific menopausal symptom domains. More rigorous trial designs incorporating active control conditions are also needed, alongside evaluation of intervention effects across different stages of menopause.

## Supplementary Information

Supplementary Material 1.

## Data Availability

All data were obtained from publicly available primary studies. For further details regarding the data used in the analysis, please contact the corresponding author.

## Abbreviations

CBT: Cognitive Behavioural Therapy
HF/NS: Hot Flushes/Night Sweats
MBSR: Mindfulness-Based Stress Reduction
MHT: Menopausal Hormone Therapy
NICE: National Institute for Health and Care Excellence
RCTs: Randomised Controlled Trials
VMS: Vasomotor Symptoms
