# Supplementary material for: The effectiveness of psychosocial interventions on physiological symptoms of menopause: a systematic review and meta-analysis
Source: BMC Womens Health. 2026 Jun 8;26:420. doi: 10.1186/s12905-026-04563-3 (PMC13491826; doi:10.1186/s12905-026-04563-3)
Supplement: Supplementary file 1 [file 12905_2026_4563_MOESM1_ESM.docx]

# Supplementary Material

Appendix 1: Search Terms for each database

**PsycINFO**

| Set | Search Statement |
| --- | --- |
| 1. | exp Randomized Controlled Trials/ |
| 2. | exp Cognitive Behavior Therapy/ or exp Behavior Therapy/ or exp Cognitive Therapy/ or exp "Acceptance and commitment therapy"/ or exp Relaxation therapy/ or exp Psychosocial Interventions/ or exp Psychotherapy/ |
| 3. | exp Menopause/ |
| 4. | (Menopaus* or peri-menopaus* or perimenopaus* or premenopaus* or post-menopaus* or postmenopaus* or climacter* or mid* age* or mid* life or midlife).ab,id,ti. |
| 5. | ("Psycho* therap*" or Psychotherap* or "Psycho* Intervention*" or "Psycho* Treatment*" or "Psychosocial intervention*" or "Psychosocial Treatment*" or "Psychosocial therap*" or "Cognitive behavio* therap*" or "Cognitive behavio* intervention*" or "Cognitive Therap*" or "Behavio* Therap*" or "group therap*" or "group intervention*" or "Mindfulness" or "Mindfulness based stress reduction" or "Mindfulness based intervention" or "Mindfulness training" or "Acceptance and commitment therapy" or Relax* or "Applied relaxation" or "Compassion focused therapy").ab,id,ti. |
| 6. | ("Menopaus* Symptom*" or "Climacteric Symptom*" or "Vasomotor Symptom*" or "Physi* symptom*" or "Vasomotor" or "Hot Fl?sh*" or "Night Sweat*" or sweat* or Sleep* or Fatigue or Insomnia or "Sexual function*" or Libido or Urogenital or vaginal or "Somatic Symptom*" or pain or ache* or headache*).ab,id,ti. |
| 7. | (Randomi?ed control* trial* or randomi?ed clinical trial* or RCT or randomi?ed control* design).ab,id,ti. |
| 8. | 1 or 7 |
| 9. | 2 or 5 |
| 10. | 3 or 4 |
| 11. | 6 and 8 and 9 and 10 |

**Medline**

| Set | Search Statement |
| --- | --- |
| 1. | exp Menopause, Premature/ or exp Menopause/ |
| 2. | psychotherapy/ or behavior therapy/ or cognitive behavioral therapy/ or exp "acceptance and commitment therapy"/ or exp mindfulness/ or exp relaxation therapy/ or psychosocial intervention/ |
| 3. | Randomized Controlled Trials as Topic/ |
| 4. | Climacteric/ |
| 5. | 1 or 4 |
| 6. | (Menopaus* or peri-menopaus* or perimenopaus* or premenopaus* or post-menopaus* or postmenopaus* or climacter* or mid* age* or mid* life or midlife).ab,kf,ti. |
| 7. | ("Psycho* therap*" or Psychotherap* or "Psycho* Intervention*" or "Psycho* Treatment*" or "Psychosocial intervention*" or "Psychosocial Treatment*" or "Psychosocial therap*" or "Cognitive behavio* therap*" or "Cognitive behavio* intervention*" or "Cognitive Therap*" or "Behavio* Therap*" or "group therap*" or "group intervention*" or "Mindfulness" or "Mindfulness based stress reduction" or "Mindfulness based intervention" or "Mindfulness training" or "Acceptance and commitment therapy" or Relax* or "Applied relaxation" or "Compassion focused therapy").ab,kf,ti. |
| 8. | ("Menopaus* Symptom*" or "Climacteric Symptom*" or "Vasomotor Symptom*" or "Physi* symptom*" or "Vasomotor" or "Hot Fl?sh*" or "Night Sweat*" or sweat* or Sleep* or Fatigue or Insomnia or "Sexual function*" or Libido or Urogenital or vaginal or "Somatic Symptom*" or pain or ache* or headache*).ab,kf,ti. |
| 9. | (Randomi?ed control* trial* or randomi?ed clinical trial* or RCT or randomi?ed control* design).ab,kf,ti. |
| 10. | 3 or 9 |
| 11. | 5 or 6 |
| 12. | 2 or 7 |
| 13. | 8 and 10 and 11 and 12 |

**Embase**

| Set | Search Statement |
| --- | --- |
| 1. | (Menopaus* or peri-menopaus* or perimenopaus* or premenopaus* or post-menopaus* or postmenopaus* or climacteric or "climacter* syndrome*" or mid* age or mid* life).ab,kf,ti. |
| 2. | ("Psycho* therap*" or Psychotherap* or "Psycho* Intervention*" or "Psycho* Treatment" or "Psychosocial intervention" or "Psychosocial Treatment" or "Cognitive behavio* therap*" or "Cognitive behavio* intervention" or "Cognitive Therap*" or "Behavio* Therap*" or "group therap*" or "group intervention" or "Mindfulness" or "Mindfulness based stress reduction" or "Mindfulness based intervention" or "Mindfulness-based stress reduction" or "Mindfulness-based intervention" or "Mindfulness training" or "Acceptance and commitment therapy" or Relax* or "Applied relaxation" or "Compassion focused therapy").ab,kf,ti. |
| 3. | ("Menopaus* Symptom" or "Climacteric Symptom*" or "Vasomotor Symptom*" or "Hot Fl?sh*" or "Hot-Fl?sh*" or "Night Sweat*" or sweat* or Sleep* or "Sleep quality" or "sleep disturbance" or Fatigue or Insomnia or "Sexual function*" or libido or Urogenital or vaginal or "Somatic Symptom*" or pain or ache*).ab,kf,ti. |
| 4. | (Randomi?ed control* trial* or randomi?ed clinical trial* or RCT or RCTs or randomi?ed control* design).ab,kf,ti. |
| 5. | menopause/ or "menopause and climacterium"/ or climacterium/ or early menopause/ or postmenopause/ or premenopause/ |
| 6. | psychotherapy/ or behavior therapy/ or cognitive behavioral therapy/ or exp "acceptance and commitment therapy"/ or exp mindfulness/ or exp relaxation therapy/ or psychosocial intervention/ |
| 7. | Randomized Controlled Trials as Topic/ |
| 8. | 1 or 5 |
| 9. | 2 or 6 |
| 10. | 4 or 7 |
| 11. | 3 and 8 and 9 and 10 |

**CINAHL**

| **#** | **Query** |
| --- | --- |
| S1 | (MH "Menopause") OR (MH "Menopause, Premature") OR (MH "Premenopause") OR (MH "Perimenopause") OR (MH "Postmenopause") OR (MH "Climacteric") |
| S2 | (MH "Randomized Controlled Trials") |
| S3 | (MH "Cognitive Therapy+") OR (MH "Behavior Therapy+") OR (MH "Psychosocial Intervention") |
| S4 | “Menopaus* Symptom*” or “Climacteric Symptom*” or “Vasomotor Symptom*” or “Physi* symptom*” or “Vasomotor” or “Hot Fl?sh*” or “Night Sweat*” or sweat* or Sleep* or Fatigue or Insomnia or “Sexual function*” or Libido or Urogenital or vaginal or “Somatic Symptom*” or pain or ache* or headache* |
| S5 | Randomi?ed control* trial* or randomi?ed clinical trial* or RCT or randomi?ed control* design |
| S6 | “Psycho* therap*” or Psychotherap* or “Psycho* Intervention*” or “Psycho* Treatment*” or “Psychosocial intervention*” or “Psychosocial Treatment*” or “Psychosocial therap*” or “Cognitive behavio* therap*” or “Cognitive behavio* intervention*” or “Cognitive Therap*” or “Behavio* Therap*” or “group therap*” or “Group intervention*” or “Mindfulness” or “Mindfulness based stress reduction” or “Mindfulness based intervention” or “Mindfulness training” or “Acceptance and commitment therapy” or Relax* or “Applied relaxation” or “Compassion focused therapy” |
| S7 | Menopaus* or peri-menopaus* or perimenopaus* or premenopaus* or post-menopaus* or postmenopaus* or climacteric or “climacter* syndrome*” or mid* age* or mid* life or midlife |
| S8 | S2 OR S5 |
| S9 | S1 OR S7 |
| S10 | S3 OR S6 |
| S11 | S4 AND S8 AND S9 AND S10 |

**Web of Science

Topic (includes title, abstract, keyword plus, and author keywords)**

**Menopause or Menopausal or peri-menopausal or peri-menopause or premenopause or premenopausal or post-menopause or post-menopausal or climacteric or “Climacteric Syndrome$” or mid* life or mid*age (Topic)**

**AND**

**“Psycho* therap*” or Psychotherap* or “Psycho* Intervention$” or “Psycho* Treatment” or “Psychosocial intervention$” or “Psychosocial Treatment” or “Cognitive behavio* therap*” or “Cognitive behavio* intervention” or “Cognitive Therap*” or “Behavio* Therap*” or “group therap*” or “group intervention” or “Mindfulness” or “Mindfulness based stress reduction” or “Mindfulness based intervention” or “Mindfulness-based stress reduction” or “Mindfulness-based intervention” or “Mindfulness training” or “Acceptance and commitment therapy” or Relax* or “Applied relaxation” or “Compassion focused therapy” (Topic)**

**AND**

**“Menopause Symptom$” or “Menopausal Symptom$” or "Climacteric Symptom$” or “Vasomotor Symptom$” or “Hot Fl?sh” or “Hot-Fl?sh” or “Hot Fl?shes” or “Hot-Fl?shes” or “Night Sweat$” or sweat* or Sleep* or “Sleep quality” or “sleep disturbance$” or Fatigue or Insomnia or “Sexual function*” or libido or Urogenital or vaginal or “Somatic Symptom$” or pain or ache* (Topic)**

**AND**

**Randomi?ed control* trial$ or randomi?ed clinical trial$ or RCT or RCTs or randomi?ed control* design (Topic)**

**Cochrane**

(Menopaus* or peri-menopaus* or perimenopaus* or premenopaus* or post-menopaus* or postmenopaus* or climacter* or “climacteric syndrome” or “middle age” or mid-life or “middle life”):ti,ab,kw

AND

(Psychotherap* or Psychosocial NEXT therap* or Psychosocial NEXT intervention* or Psychosocial NEXT Treatment* or Psychological NEXT therap* or Psychological NEXT intervention** or Psychological NEXT treatment* or Cognitive NEXT Therap* or Behavio* NEXT Therap* or group NEXT therap* or Group NEXT intervention* or “Mindfulness” or “Mindfulness based stress reduction” or “Mindfulness based intervention” or “Mindfulness training” or “Acceptance and commitment therapy” or Relax* or “Applied relaxation” or “Compassion focused therapy”):ti,ab,kw

AND

((Menopaus* NEXT symptom*) or (Climacteric NEXT Symptom*) or (Vasomotor NEXT Symptom*) or (Physi* NEXT Symptom*) or Vasomotor or (Hot NEXT Flush*) or (Hot NEXT Flash*) or (Night NEXT Sweat*) or sweat* or Sleep* or Fatigue or Insomnia or (Sexual NEXT function*) or Libido or Urogenital or vaginal or (Somatic NEXT Symptom*) or pain or ache* or headache*):ti,ab,kw

AND

(Randomised NEAR/1 Trial* or Randomised NEAR/1 Design or Randomized NEAR/1 Trial* or Randomized NEAR/1 Design):ti,ab,kw

Appendix 2: Risk of Bias 2 Tool Ratings

Appendix 3: Sensitivity analysis for each outcome measure

3.1 - HF/NS Bothersomeness: Short and Medium-Term

3.2 - HF/NS Frequency: Short and Medium-Term

3.3 - Vasomotor Severity: Short-Term

3.4 - Sleep quality: Short-Term with subgroup analysis; intervention type and menopause type

3.5 Sleep quality: Medium-Term with subgroup analysis; including menopause type

3.6 – Insomnia: Short-Term with subgroup analysis on menopause type

3.7 – Insomnia: Medium-term

3.8 - Sexual functioning: Short term, with subgroup analysis on intervention type

3.9 - Sexual functioning: Medium term, with subgroup analysis on intervention type

3.10 -Fatigue: Short and medium-term

3.11-Pain: Short and medium-term

3.12- Urogenital**:** Short-term
